# Two Separable Aging Domains in Blood DNA Methylation Scores Developed for Cancer Research: Mitotic Replication and Early-Life Systemic Dysregulation

**DOI:** 10.3390/genes17091083

**Published:** 2026-09-09

**Authors:** Katelyn N. Austin, Man-Kit Lei

**Affiliations:** Department of Sociology, The University of Georgia, Athens, GA 30602, USA

**Keywords:** DNA methylation, epigenetic clocks, replication/mitotic aging, early-life systemic dysregulation, social determinants of health, cancer disparities

## Abstract

**Background/Objectives**: DNA methylation (DNAm)-based aging measures are increasingly being used in biological aging, disease vulnerability, and cancer-related risk. However, it remains unclear whether these measures capture a single generalized aging process or separable biological domains relevant to cancer-relevant biological vulnerability. **Methods**: Using data from the Family and Community Health Study (FACHS), we examine whether blood-based DNAm scores capture two distinct biological domains relevant to cancer susceptibility: a replication/mitotic domain, indexed by CellDRIFT, EpiTOC2, and MiAge, and an early-life systemic dysregulation domain, indexed by DunedinPACE, GrimAgeV2, and PhenoAge. **Results**: Regression results showed clear domain specificity. CRP, BMI, alcohol methylation, and AHRR/cg05575921 were positively associated with the early-life systemic dysregulation domain but showed weak or null associations with the replication/mitotic domain. Cross-domain tests confirmed significantly stronger associations for the early-life systemic dysregulation domain for CRP, BMI, and alcohol methylation, with a marginal difference for AHRR/cg05575921. In contrast, telomere-related methylation and immune-cell composition showed a different pattern. DNAmTL was inversely associated with both domains and did not differ significantly across domains. **Conclusions**: These findings suggest that blood-based DNAm aging measures do not reflect a single aging process. Instead, they distinguish at least two cancer-relevant methylation domains: one reflecting replication-driven mitotic aging and hematopoietic cell-turnover biology, and another reflecting early-life systemic dysregulation vulnerability linked to inflammation, metabolic risk, smoking methylation, and alcohol-related methylation. This two-domain framework may help clarify how blood DNAm profiles capture complementary biological processes relevant to cancer susceptibility and guide future studies of methylation-based cancer risk.

## 1. Introduction

Previous advances have enabled the use of epigenetic clocks—biomarkers derived from DNA methylation (DNAm) patterns—to estimate an individual’s biological age relative to chronological age [1,2]. DNAm aging measures are increasingly used as biomarkers of biological aging, disease vulnerability, and cancer-relevant biological risk. However, these clocks are often treated as interchangeable indicators of “biological aging,” even though they were developed to capture fundamentally different biological processes [3]. Since these clocks are trained on different targets, chronological-age clocks, mortality-oriented clocks, and pace-of-aging measures should not automatically be interpreted as measuring the same biology. First-generation DNAm clocks were designed primarily to estimate chronological age from methylation patterns at specific CpG sites [4]. Although first-generation clocks estimate chronological age with high accuracy, later-generation clocks—such as PhenoAge, GrimAgeV2, and DunedinPACE—were developed to capture morbidity, mortality, physiological decline, smoking history, and pace of biological aging [5]. By moving beyond strictly age-correlated CpG sites, these later-generation clocks provide a broader view of an individual’s biological state [3].

This distinction is particularly critical in oncology because DNAm loss is observed in both aged tissue and primary cancer samples. This loss may be related to the low fidelity of DNA methyltransferase (DNMT) during cell division [6]. Although DNAm loss occurs during normal aging, it appears to be accelerated or dysregulated in cancer. A separate class of DNAm measures, including CellDRIFT, EpiTOC2, and MiAge, may capture replication-driven or mitotic aging processes. Distinguishing between these mechanisms matters for cancer research because cancer susceptibility may involve both early-life systemic dysregulation vulnerabilities linked to inflammatory and exposure-related burden and replication-driven cell-turnover biology [6]. However, few studies have directly tested whether blood-based DNAm scores reflect general aging signals or multiple separable biological domains [7]. To address this gap, the present study tests whether blood-based DNAm scores can successfully distinguish between two domains: a replication/mitotic methylation domain, reflecting cellular division (cumulative cell division, hematopoietic turnover, methylation drift, and clonal expansion) and an early-life systemic dysregulation domain that reflects systemic wear-and-tear associated with life exposures and stress (inflammation, metabolic dysregulation, smoking, alcohol exposure, and multisystem wear).

### 1.1. Replication/Mitotic Methylation Domain

Since aging is the key risk factor for disability and diseases such as cancer, measures of biological age and rate of aging at the molecular level are important [7,8]. Past research has found that age-related accumulation of somatic mutations drives age-related cancer, but it does not fully capture the story. More recently, aging and cancer were shown to share a common epigenetic replication signature termed CellDRIFT [8]. CellDRIFT is a methylation-based correlate of cellular replicative history. Over time, repeated cell division can produce cumulative mistakes, leading to replication-associated epigenetic wear and tear. CellDRIFT increases with age across multiple tissues and can distinguish tumors from normal tissue; it has been analyzed in cancers of the thyroid, breast, lung, pancreas, and colon. A significant increase in CellDRIFT was found in these tumor samples compared to corresponding normal tissues [8]. These findings suggest that age-related replication may drive epigenetic changes in cells and could push them towards a more tumorigenic state, meaning that aging and cell division may contribute to cancer not just by breaking down the DNA, but by slowly corrupting the epigenetics.

Given that many cancer risk factors have been associated with DNAm changes in normal cells at the same sites that undergo DNAm and changes with age in healthy tissue, DNAm-based mitotic clocks potentially could predict cancer risk [9]. A DNAm-based age-correlative model called EpiTOC (Epigenetic Timer of Cancer) correlates its tick-rate predictor with the rate of stem cell division in normal tissues, as well as to an mRNA expression-based mitotic index in cancer tissue [9]. Unlike other epigenetic clocks, the tick rate of EpiTOC is universally accelerated in cancer, preinvasive lesions, in normal epithelial cells at risk of neoplastic transformations, and in normal epithelial cells exposed to smoke carcinogens. Since cell proliferation is significant in cancer outcomes, it is reasoned that EpiTOC was expected to show an accelerated tick rate across cancer types, which was confirmed [9]. Unlike other mutational clock-like signatures, EpiTOC provides concrete evidence of a molecular mitotic-like clock that shows universal acceleration in cancer. Altogether, EpiTOC may constitute an informative biomarker of cancer risk if evaluated in the cell of origin or in a relevant surrogate tissue.

Another piece of the domain that allows a quantitative estimate of mitotic age, or the total number of lifetime cell divisions in a tissue, is the MiAge model. The MiAge domain showed that across the thirteen cancer types, the estimated mitotic age was universally accelerated in tumor tissues compared to adjacent normal tissues [10]. Overall, this domain is expected to reflect cumulative cellular replication, mitotic history, hematopoietic cell turnover, telomere-related cellular aging, and immune-cell composition differences. This is relevant to cancer due to the fact that repeated cell division increases opportunities for replication errors, methylation drift, and clonal expansion [11,12]. This domain may capture immune-cell turnover and proliferative history in hematopoietic systems [13].

### 1.2. Early-Life Systemic Dysregulation Domain

Phenotypic DNAm clocks provide an alternative approach to measuring age-related deterioration and mortality [14]. These clocks use biological and physiological biomarkers to quantify aging and disease-related mortality. They have also shown greater accuracy in predicting mortality than chronological age. Three notable phenotypic clocks are PhenoAge [15], GrimAge [16], and DunedinPoAm [17]. PhenoAge was derived by linking DNAm patterns to a clinical phenotypic age measure based on chronological age and nine biomarkers, whereas GrimAge was trained on DNAm surrogates of plasma proteins and smoking exposure to predict mortality risk [14]. DunedinPoAm, later refined as DunedinPACE, was developed from longitudinal changes in 18 biomarkers to estimate the pace of aging.

This domain is hypothesized to capture early-life systemic dysregulation rather than cumulative mitotic or replication history. In blood, these DNAm measures may reflect inflammatory burden, metabolic dysregulation, smoking- and alcohol-related exposures, and broader multisystem wear-and-tear that together shape the biological environment in which cancer risk emerges. This distinction is important because cancer susceptibility may depend not only on cellular replication and epigenetic drift, but also on systemic conditions that promote tumor initiation or progression. Accordingly, biomarkers such as CRP, BMI, alcohol methylation, and AHRR/cg05575921 are expected to align more strongly with the early-life systemic dysregulation domain than with the replication/mitotic domain.

### 1.3. Blood-Based DNAm Measures in Cancer

Blood DNAm markers are promising tools for cancer research, but their associations with cancer risk are often modest, heterogeneous, and partly confounded by immune-cell composition [18,19]. Blood-based DNAm aging measures appear to capture cancer-relevant biological signals, but their effect sizes and interpretations vary across clocks and malignancies [19]. The strongest prospective associations have often been reported for GrimAge and other exposure-linked clocks, particularly in relation to lung cancer, whereas associations for other DNAm markers have been less consistent across cancer outcomes [18,20]. Associations of blood-based mitotic clocks with cancer risk may also be partly explained by immune-cell heterogeneity, suggesting that these measures may reflect a combination of proliferative history, hematopoietic turnover, and leukocyte composition [18,21].

Despite the existence of the abovementioned literature, few studies have tested whether cancer-relevant blood DNAm scores are better represented by one general aging signal or multiple separable biological domains. This distinction is important because the present study compares measures hypothesized to reflect replication and mitotic biology with measures hypothesized to reflect an early-life systemic dysregulation vulnerability [18,22]. The present study also does not estimate cancer incidence or distinguish between cancer types. Its cancer relevance is therefore mechanistic and inferential: the replication/mitotic domain is connected to proliferative and hematopoietic biology, whereas the early-life systemic dysregulation vulnerability domain is connected to inflammatory, metabolic, smoking-related, and multisystem processes that may shape the context in which carcinogenesis occurs. These domains should therefore not be interpreted as equivalent to tumor-derived biomarkers or validated cancer predictor scores.

### 1.4. The Current Study

In the current study, blood-based DNAm aging scores may not represent a single biological aging process. Instead, they may organize into partially distinct domains: one related to replication/mitotic and hematopoietic cell-turnover biology, and another related to early-life systemic dysregulation vulnerability and exposure-related inflammatory burden. Distinguishing these domains could clarify how blood-based aging measures should be interpreted in cancer and social determinants research. To evaluate this possibility, the study tests five formal hypotheses. First, CellDRIFT, EpiTOC2, and MiAge are expected to be positively correlated and to load together on a common replication/mitotic DNAm domain. Second, PhenoAge, GrimAgeV2, and DunedinPACE are expected to be positively correlated and to load together on an early-life systemic dysregulation vulnerability domain. Third, replication/mitotic scores are expected to show weak, null, or negative associations with the early-life systemic dysregulation score, indicating that the two domains are not interchangeable. Fourth, CRP, BMI, alcohol methylation, and AHRR/cg05575921 are expected to show stronger associations with the early-life systemic dysregulation vulnerability domain than with the replication/mitotic domain. Fifth, DNAmTL and immune-cell composition are expected to show stronger associations with the replication/mitotic domain than with the early-life systemic dysregulation vulnerability domain. Using blood DNAm data from 420 participants in the Family and Community Health Study, we compare a one-factor model with a correlated two-factor model and examine whether inflammatory, exposure-related, telomere, and immune-cell indicators show domain-specific associations.

## 2. Materials and Methods

### 2.1. Study Participants

We used data from the Family and Community Health Study (FACHS), a longitudinal study of Black families in Georgia and Iowa. FACHS began in 1997–1998 with 889 target youths who were in the fifth grade at the time of recruitment. FACHS interviewed the target youth and a primary caregiver from the first wave onward. Details regarding recruitment and the study design have been reported elsewhere [23,24]. The current study uses data from the target participants at Wave 7. Participants were re-interviewed in 2015–2016, when they were, on average, 28.67 years old (SD = 0.79). At Wave 7, 449 targets had valid blood-based methylation data [25]. We used these cases as the analytic frame and merged the files containing the methylation scores, exposure measures, CRP, BMI, sex, and estimated immune-cell proportions by case identifier.

We next restricted the analysis to participants with complete data on the six DNAm scores, the four exposure and inflammatory measures, DNAm-estimated telomere length, the five estimated immune-cell proportions, age, and sex. Twenty-nine of the 449 participants were missing at least one exposure or inflammatory measure and were excluded. No further cases were lost because of missing DNAm scores, DNAmTL, immune-cell estimates, age, or sex. The final analytic sample included 420 young adults (see Table A1): 158 men (37.6%) and 262 women (62.4%). To evaluate selection into the analytic sample, we compared these 420 participants with the 469 baseline targets who were not included using baseline age, sex, state, family structure, poverty, and parental education (Table A2). We also used these baseline measures to construct inverse-probability-of-selection weights for a sensitivity analysis.

### 2.2. Procedures and DNA Methylation Assessment

The FACHS protocol and all study procedures were approved by the University of Georgia Institutional Review Board (Protocol 00004856). Computer-assisted interviews were administered in respondents’ homes and took, on average, about two hours. The interviewer read each question aloud, and the respondent entered answers on a separate keypad. At Wave 7, the interview and blood collection were completed within one week of one another. Blood was processed into DNA and serum. DNA was stored at −20 °C and serum at −80 °C until laboratory analysis [26]. Whole-blood DNA was prepared using cold protein precipitation and quantified with a NanoDrop photometer (Thermo Fisher Scientific, Waltham, MA, USA) [27].

Genome-wide DNAm was assessed with the Illumina Infinium HumanMethylationEPIC BeadChip. Participants were distributed across assay slides, and replicate DNA samples were included to monitor batch variation. Type I and Type II probes were quantile normalized separately. For each CpG site, the beta value was calculated as the methylated-probe intensity divided by the sum of the methylated and unmethylated probe intensities. The normalized methylation data were then used to calculate the six DNAm scores and DNAm-estimated telomere length with the methylCIPHER package v0.2.0 [28]. The test procedures are described in detail in Appendix A.

### 2.3. Measures

#### 2.3.1. Replication/Mitotic DNAm Scores

We assessed replication/mitotic aging with CellDRIFT, the Epigenetic Timer of Cancer version 2 (EpiTOC2), and mitotic age (MiAge). These measures were developed to capture methylation changes associated with repeated cell division. CellDRIFT was derived from changes observed during extensive cellular passaging and is elevated in tumor tissue and in some normal tissues from individuals with cancer [8]. EpiTOC2 uses methylation at polycomb target sites to estimate stem-cell division rate and was developed for research on mitotic activity and cancer risk [29]. MiAge estimates the mitotic age of human tissues from DNAm patterns [10]. Higher values on each measure indicate greater replication-related or mitotic aging.

#### 2.3.2. Early-Life Systemic Dysregulation Vulnerability DNAm Scores

We assessed early-life systemic dysregulation vulnerability with PhenoAge, GrimAge version 2 (GrimAgeV2), and DunedinPACE. PhenoAge was trained to reproduce a clinical phenotypic-age measure associated with morbidity and mortality [15]. GrimAgeV2 combines DNAm surrogates for smoking and plasma proteins to predict mortality and age-related disease risk [30]. DunedinPACE was trained on longitudinal change in multiple organ systems and estimates the pace of physiological decline [31]. Although the three measures differ in their construction, each was designed to capture health span, mortality risk, or multisystem aging rather than the cumulative history of cell division.

#### 2.3.3. Cancer-Relevant Exposures and Inflammation

We examined four cancer-relevant exposure and inflammatory measures. CRP was obtained from the Wave 7 serum laboratory file and served as a marker of systemic inflammation. Serum was stored at −80 °C until analysis [26]. Serum CRP was measured in mg/L using an enzyme-linked immunosorbent assay from R&D Systems [32]. BMI was calculated from measured height and weight. Smoking exposure was assessed by methylation at AHRR/cg05575921 using a methylation-sensitive droplet digital PCR assay [33]. Because lower methylation at this locus indicates greater cigarette exposure, we multiplied the raw value by −1 before standardization; higher scores therefore indicate greater smoking-related exposure. Alcohol exposure was assessed with the Alcohol T Score, a whole-blood methylation score based on four loci associated with heavy alcohol consumption [34,35,36]. Higher values indicate a stronger methylation signature of alcohol exposure.

#### 2.3.4. DNAm Telomere Length

DNAm-estimated telomere length was calculated from the normalized methylation data using the estimator developed in [16]. This measure predicts leukocyte telomere length from methylation patterns. Higher values indicate longer estimated telomere length.

#### 2.3.5. Immune-Cell Composition

We used methylation-estimated proportions of CD8+ T cells, CD4+ T cells, natural killer cells, B cells, and monocytes. The estimates were obtained from whole-blood EPIC-array data using a Houseman-type reference-based deconvolution procedure with an EPIC-optimized IDOL library [37,38,39]. Because these variables represent parts of blood-cell composition, they are not statistically independent. Accordingly, coefficients from models that include all five cell estimates are interpreted as conditional associations, net of the other included cell fractions, rather than as effects of isolated cell populations. Because the full set of blood-cell fractions sums to one, an increase in one cell fraction must be accompanied by a decrease in one or more other fractions. Therefore, the estimated cell fractions are not independent. Coefficients from models that include all five cell estimates show the association for each cell fraction after accounting for the other included fractions. They should not be interpreted as the independent effect of an individual cell type.

#### 2.3.6. Covariates

All regression models controlled for chronological age, age squared, and sex. Sex was coded 1 for women and 0 for men.

### 2.4. Analytic Strategy

All analyses were conducted in Stata 19. We first verified that the 449-person methylation file remained unchanged across the auxiliary-file merges and then applied the complete-case restriction described above. The six DNAm scores and all continuous predictors were standardized after the restriction. Thus, each focal regression coefficient represents the standard-deviation difference in the outcome associated with a one-standard-deviation difference in the predictor, with the other variables in the equation held constant.

We began by examining kernel-density plots and bivariate Pearson correlations among the six scores. We then submitted the standardized scores to an unrotated principal component analysis based on the correlation matrix. Principal component loading figure plots the correlations between each score and the first two components, calculated by multiplying each component eigenvector by the square root of its eigenvalue. The sign of a principal component is arbitrary; therefore, the analysis focuses on the clustering and separation of the scores rather than the direction of the component axes.

We next estimated a one-factor confirmatory model as a reference and compared it with a correlated two-factor model. The one-factor model specified all six scores as indicators of one general DNAm aging factor. The two-factor model specified CellDRIFT, EpiTOC2, and MiAge as indicators of a replication/mitotic factor and PhenoAge, GrimAgeV2, and DunedinPACE as indicators of an early-life systemic dysregulation vulnerability factor. The unconstrained models placed the residual variance of MiAge at the boundary of zero. We therefore fixed the MiAge residual variance at 0.01 in both models. Applying the same small residual constraint to both models permitted a common comparison while making the boundary condition explicit. The value of 0.01 is a small positive constant chosen only to move the estimate off the zero boundary; because the primary domain scores are equally weighted composites rather than factor scores, the substantive results do not depend on the exact value of this constraint. We evaluated model fit with the chi-square statistic, comparative fit index (CFI), Tucker–Lewis index (TLI), root mean square error of approximation (RMSEA), standardized root mean square residual (SRMR), Akaike information criterion (AIC), and Bayesian information criterion (BIC). We interpreted these indices with reference to established cutoffs for approximate fit [40].

The primary domain scores were not estimated from the CFA. Instead, they were formed a priori as equally weighted composites. We averaged standardized CellDRIFT, EpiTOC2, and MiAge values and standardized the resulting mean to form the replication/mitotic score. We followed the same procedure with PhenoAge, GrimAgeV2, and DunedinPACE to form the early-life systemic dysregulation vulnerability score. This approach gave each measure equal weight and did not allow sample-specific factor loadings to determine the primary outcomes. We report Cronbach’s alpha as a descriptive index of within-domain coherence and the correlation between the two composite scores as an indication of their overlap.

We estimated two sets of linear regression models with heteroskedasticity-robust standard errors. The first set included CRP, BMI, the reversed AHRR/cg05575921 smoking measure, and the alcohol methylation score in the same equation. The second set included DNAmTL and all five estimated immune-cell proportions. Each model controlled for mean-centered age, centered age squared, and sex. We examined variance inflation factors after estimation. Because the two domain outcomes were measured in the same participants, we used seemingly unrelated estimation to test whether each predictor’s coefficient differed across domains. The tabulated coefficients and confidence intervals come from the single-equation models with heteroskedasticity-robust standard errors, whereas the cross-domain equality tests come from seemingly unrelated estimation, which accounts for the covariance between the two coefficient estimates obtained from the same individuals; the point estimates are identical under both approaches. We conducted 10 cross-domain tests and report both unadjusted *p* values and Benjamini–Hochberg false-discovery-rate adjusted q values [41]. False-discovery-rate control was applied to this primary set of cross-domain tests; the component-level, leave-one-score-out, and single-cell models reported below are descriptive sensitivity analyses and were not included in this correction.

We also checked whether the findings depended on how the two domains were scored or on who remained in the analytic sample. First, we used standardized factor scores from the constrained two-factor CFA and reran the regression and cross-domain tests. We then estimated each participant’s probability of inclusion in the 420-person sample using baseline age, sex, state, family structure, poverty, parental education, and missing poverty status. The models were repeated using the resulting stabilized inverse-probability-of-selection weights. These weighted models were used to examine whether the coefficient pattern changed after adjustment for the measured baseline differences between included and nonincluded participants. They were not treated as proof that all sources of selection had been removed [42]. Because Stata’s seemingly unrelated estimation procedure does not accept pweighted regression estimates, the weighted sensitivity analysis focused on the direction, magnitude, and statistical precision of the pweighted coefficients rather than on a second set of formal cross-domain equality tests.

Finally, we estimated the exposure and inflammatory model separately for each of the six component scores and reconstructed the early-life systemic dysregulation composite after omitting one score at a time. We also residualized each DNAm score for linear and quadratic age and repeated the correlation and principal component analyses. For the cell-composition analyses, we estimated separate models for each cell proportion together with DNAmTL and the covariates. These models do not remove the compositional dependence among blood-cell fractions, but they show whether the sign of an association depends on simultaneous adjustment for the other cell estimates.

Because CellDRIFT, EpiTOC2, and MiAge were highly correlated, we conducted an additional sensitivity analysis treating them as a single replication/mitotic measure. We formed a standardized equal-weight composite of the three scores and examined its correlations with PhenoAge, GrimAgeV2, and DunedinPACE. We then repeated the PCA using the mitotic composite and the three early-life systemic dysregulation measures to determine whether the separation remained after reducing the three highly correlated mitotic scores to one measure.

## 3. Results

### 3.1. Sample Construction, Selection, and Score Distributions

FACHS began with 889 target youths and their families. By Wave 7, 449 targets had valid EPIC-array methylation data. We used the 449 methylation cases as the starting sample. Twenty-nine were then excluded because one or more focal exposure or inflammatory measures were missing. Three did not match the CRP file, and 26 were missing at least one focal measure after the files were merged. No further cases were lost because of missing DNAm scores, age, sex, DNAmTL, or estimated immune-cell proportions. The final analytic sample included 420 participants, representing 47.2% of the original FACHS targets (Table A1).

Table A2 compares the 420 participants in the analytic sample with the 469 who were not included. Those included were slightly younger at Wave 1 than those not included (10.95 versus 11.07 years, *p* = 0.003). They were also less likely to be male (37.6% versus 53.9%, *p* < 0.001) and less likely to have been recruited in Iowa (39.8% versus 64.0%, *p* < 0.001). The groups did not differ significantly in household poverty, parental education below high school, family structure, or missing baseline poverty information. The largest standardized differences were for state (0.48) and sex (0.33).

**Figure 1:**
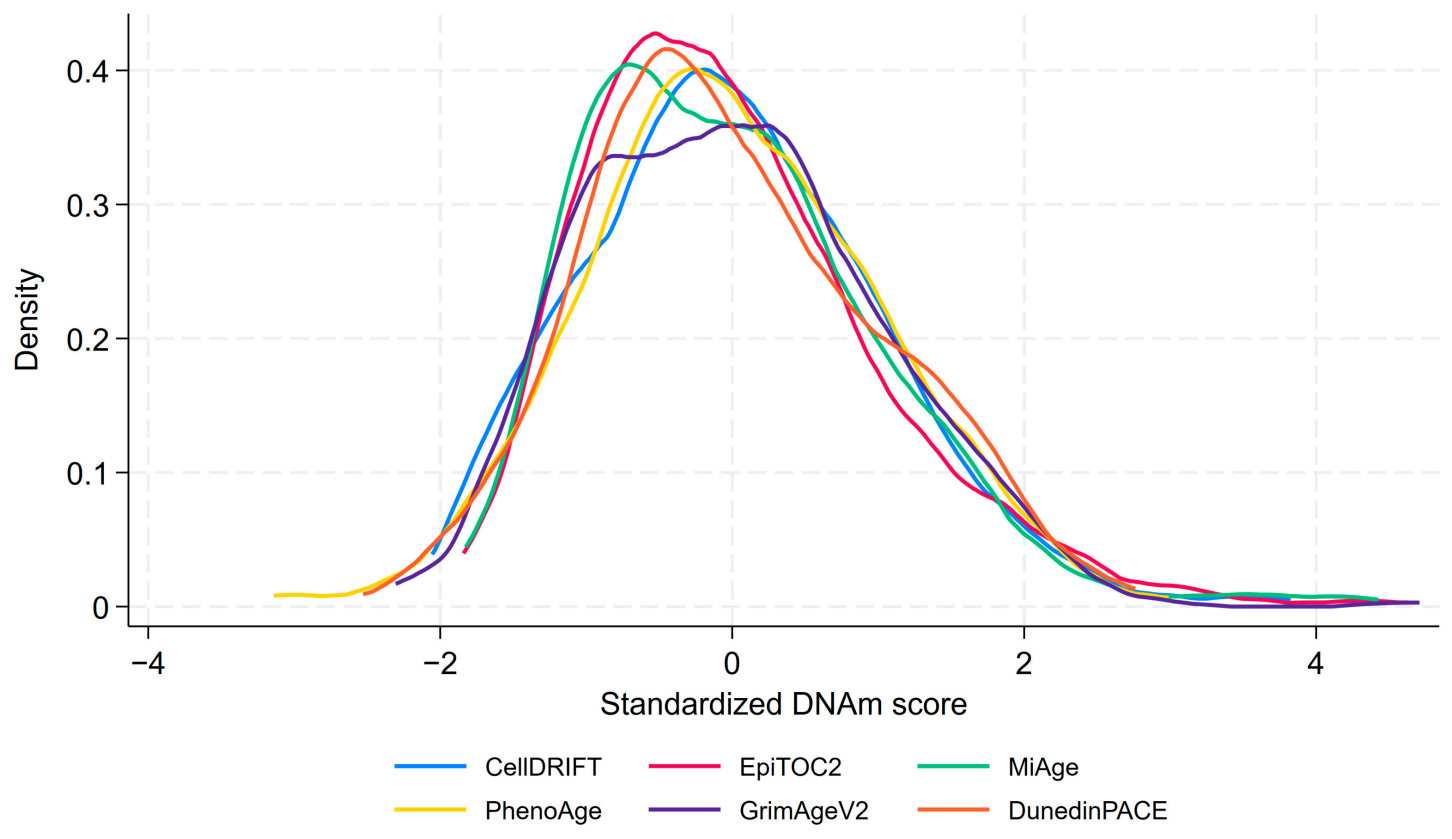
Kernel-density distributions of the six standardized blood DNAm scores. All scores were standardized within the complete analytic sample (*N* = 420). Each measure had a single main peak, although the height of the peak and the length of the upper tail differed across scores.

The selection model produced stabilized weights with a mean of 0.99, a standard deviation of 0.37, and a range from 0.61 to 2.98. Thus, there was no evidence of extremely large weights. Results from the weighted models are reported below with the other sensitivity analyses. We then examined the distributions of the six standardized DNAm scores. Figure 1 shows that each measure was continuous and had a single main peak. The scores differed in the height of the peak and the length of the upper tail, but none showed a separate mode or a break in the distribution. We retained all six measures in continuous form.

### 3.2. Correlations, Principal Components, and Confirmatory Factor Analysis

Table 1 presents the zero-order correlations among the six DNAm scores. CellDRIFT, EpiTOC2, and MiAge were strongly related. CellDRIFT correlated 0.711 with EpiTOC2 and 0.863 with MiAge, and EpiTOC2 correlated 0.842 with MiAge (all *p* < 0.01). The three early-life systemic dysregulation measures were also positively related, although the correlations were smaller. PhenoAge correlated 0.449 with GrimAgeV2 and 0.492 with DunedinPACE, and GrimAgeV2 correlated 0.643 with DunedinPACE (all *p* < 0.01).

Correlations across the two sets were small. PhenoAge was weakly and inversely associated with CellDRIFT (r = −0.099, *p* = 0.043) and was unrelated to EpiTOC2 and MiAge. Correlations of GrimAgeV2 with the replication/mitotic measures ranged from −0.066 to −0.082 and did not reach *p* < 0.05. DunedinPACE was inversely associated with CellDRIFT (r = −0.131, *p* = 0.007), EpiTOC2 (r = −0.118, *p* = 0.016), and MiAge (r = −0.105, *p* = 0.032). The correlations were therefore large within the replication/mitotic set, moderate within the early-life systemic dysregulation set, and near zero across the two sets.

**Figure 2:**
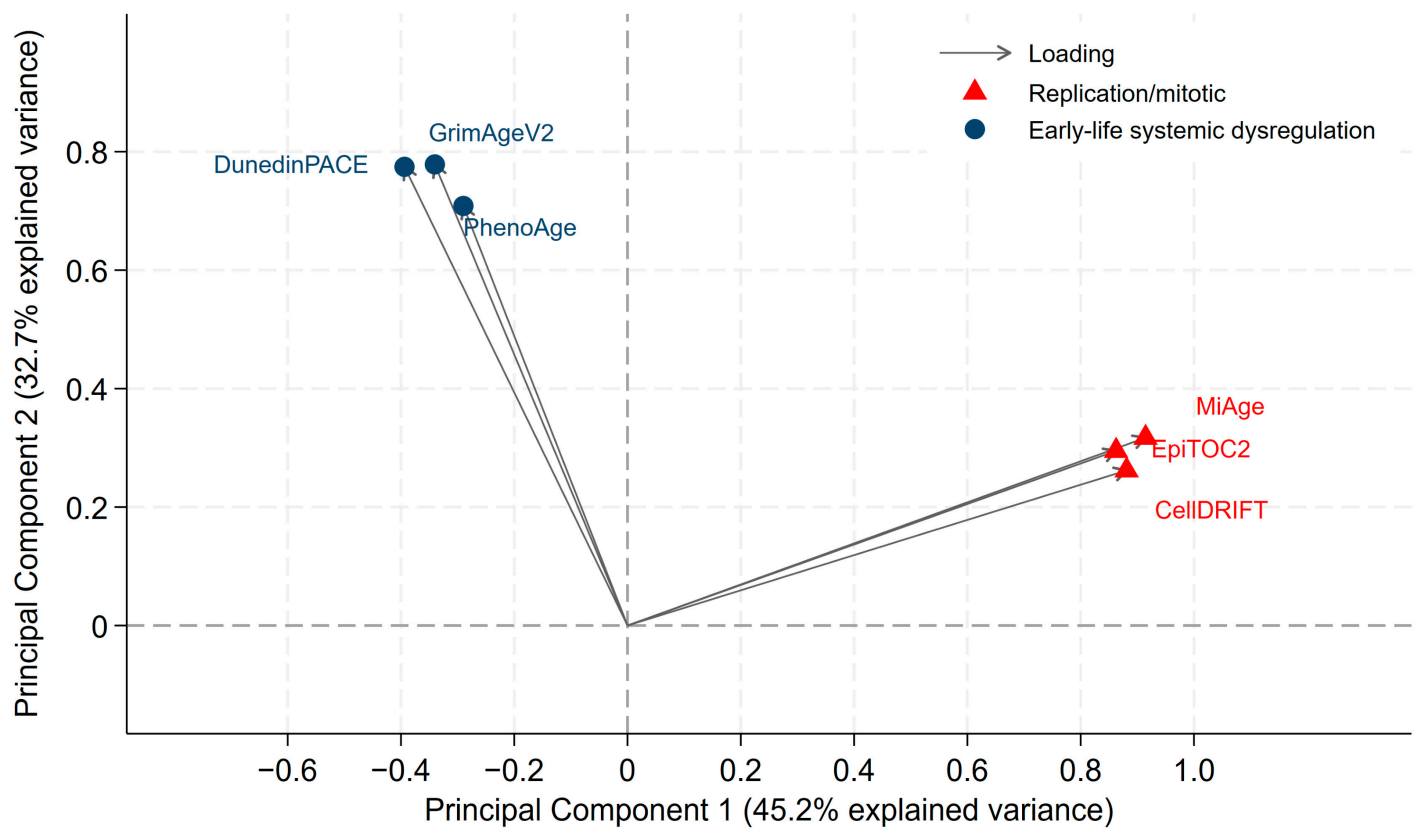
Principal component loading plot for the six standardized DNAm scores. The plotted coordinates are correlations between each score and the first two components. CellDRIFT, EpiTOC2, and MiAge clustered together, whereas PhenoAge, GrimAgeV2, and DunedinPACE occupied a separate region of the plot. Component 1 explained 45.2% of the variance and Component 2 explained 32.7%; together they explained 77.9%. The confirmatory factor analysis reported in Table 2 provided a separate test of this two-domain structure.

The principal component results show the same separation (Figure 2). The first component had an eigenvalue of 2.712 and accounted for 45.2% of the variance. CellDRIFT, EpiTOC2, and MiAge loaded 0.881, 0.863, and 0.915 on this component. PhenoAge, GrimAgeV2, and DunedinPACE loaded in the opposite direction, with loadings of −0.290, −0.340, and −0.394. The second component had an eigenvalue of 1.964 and explained another 32.7% of the variance. PhenoAge, GrimAgeV2, and DunedinPACE loaded 0.708, 0.778, and 0.774 on this component, whereas the replication/mitotic loadings ranged from 0.262 to 0.317. Together, the first two components explained 77.9% of the variation in the six scores.

The CFA results are shown in Table 2. The one-factor model fit poorly: chi-square (10) = 370.38, *p* < 0.001, CFI = 0.752, TLI = 0.628, RMSEA = 0.293, and SRMR = 0.201. In that model, CellDRIFT, EpiTOC2, and MiAge loaded 0.866, 0.846, and 0.995, whereas PhenoAge, GrimAgeV2, and DunedinPACE loaded −0.037, −0.083, and −0.108. The single factor did not represent a general aging signal. It reproduced the replication/mitotic measures and left the early-life systemic dysregulation measures essentially unmodeled. The correlated two-factor model fit the data much better: chi-square (9) = 17.35, *p* = 0.044, CFI = 0.994, TLI = 0.990, RMSEA = 0.047, and SRMR = 0.019. The RMSEA 90% confidence interval ranged from 0.008 to 0.080, and the test of close fit was not rejected (pclose = 0.510). The AIC and BIC were lower for the two-factor model by 351.0 and 347.0 points, respectively. Taken together, the fit indices do not support a single common factor. The two-factor model reproduced the observed covariance structure much more closely.

In the two-factor model, CellDRIFT, EpiTOC2, and MiAge loaded 0.866, 0.846, and 0.995, respectively, on the replication/mitotic factor. The MiAge loading was nearly one because its residual variance was fixed at 0.01 after the unconstrained estimate reached zero. PhenoAge, GrimAgeV2, and DunedinPACE loaded 0.584, 0.764, and 0.842 on the early-life systemic dysregulation vulnerability factor. The two factors were weakly and negatively correlated (r = −0.114, *p* = 0.035). Although the CFA supported the two-domain structure, the boundary estimate for MiAge limits how strongly this model should be interpreted. The three replication/mitotic scores correlated between 0.711 and 0.863 and produced an alpha of 0.926, so in whole blood they behave less like three indicators of a domain than like three measurements of one construct. For this reason, we used the equal-weight composites as the primary outcomes.

The replication/mitotic measures had a Cronbach’s alpha of 0.926. Alpha was lower for the early-life systemic dysregulation measures (0.770), consistent with their more moderate intercorrelations. After the two equal-weight composites were formed, the correlation between them was small and negative (r = −0.108, *p* = 0.028). The correlation was nearly the same as the latent-factor correlation.

### 3.3. Exposure and Inflammatory Predictors

**Figure 3:**
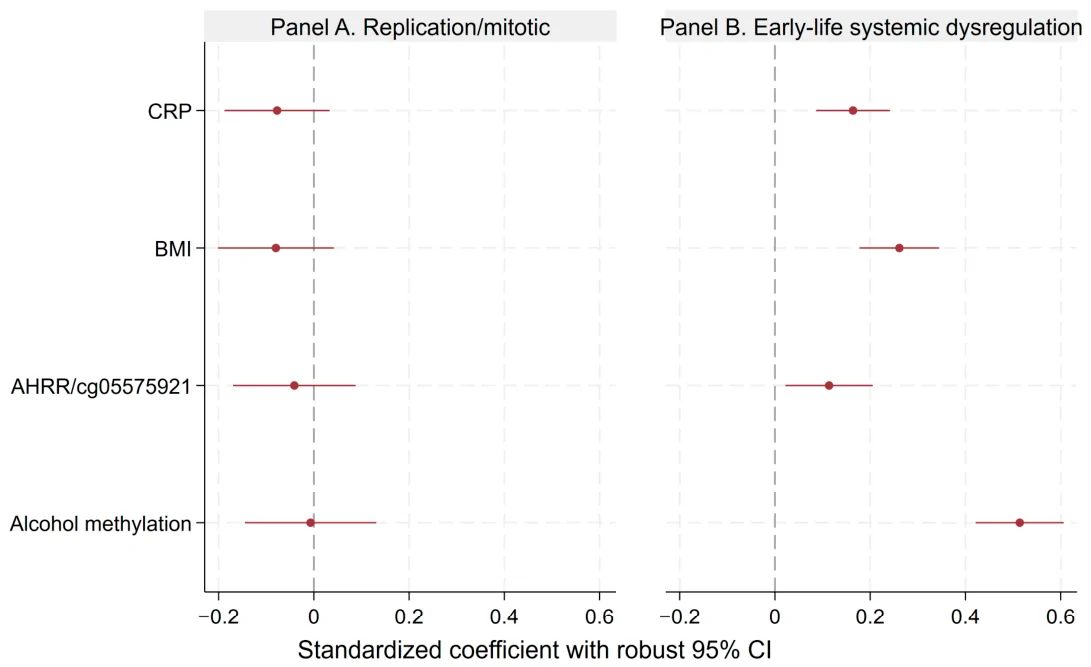
Associations of CRP, BMI, AHRR/cg05575921, and alcohol methylation with the replication/mitotic and early-life systemic dysregulation vulnerability domains. Points are standardized regression coefficients, and horizontal lines are heteroskedasticity-robust 95% confidence intervals. Each model includes all four focal predictors and adjusts for mean-centered age, centered age squared, and sex. CRP, BMI, and alcohol methylation were associated with early-life systemic dysregulation vulnerability but not with replication/mitotic aging.

Table 3 and Figure 3 show the associations of CRP, BMI, AHRR/cg05575921, and alcohol methylation with the two domain scores. Turning first to replication/mitotic aging, the full model explained 1.7% of the variance and was not statistically significant (*p* = 0.282). None of the four predictors reached *p* < 0.05 for the replication/mitotic domain: CRP (b = −0.077, 95% CI [−0.188, 0.033]), BMI (b = −0.080, 95% CI [−0.201, 0.042]), the AHRR smoking measure (b = −0.041, 95% CI [−0.170, 0.088]), and alcohol methylation (b = −0.007, 95% CI [−0.145, 0.131]). All four confidence intervals included zero, although they remain compatible with inverse associations of about 0.2.

The findings were quite different for early-life systemic dysregulation vulnerability. The model explained 47.8% of the variance. CRP was positively associated with the domain (b = 0.164, 95% CI [0.086, 0.242], *p* < 0.001), as was BMI (b = 0.261, 95% CI [0.178, 0.345], *p* < 0.001). The reversed AHRR/cg05575921 measure also had a positive coefficient (b = 0.114, 95% CI [0.022, 0.205], *p* = 0.015). Alcohol methylation showed the largest association (b = 0.514, 95% CI [0.421, 0.607], *p* < 0.001). The Alcohol T score was measured using MSdPCR rather than derived from the EPIC array used to calculate the aging measures. However, because both the predictor and outcome are DNAm-based, the association may partly reflect shared methylation biological and should not be interpreted as fully independent biological validation. Because the variables were standardized, this coefficient indicates that a one-standard-deviation increase in the alcohol methylation score was associated with slightly more than one-half of a standard deviation higher early-life systemic dysregulation vulnerability, net of CRP, BMI, AHRR/cg05575921, age, age squared, and sex.

We next tested whether these coefficients differed across the two outcomes. The early-life systemic dysregulation coefficient was significantly larger than the replication/mitotic coefficient for CRP (*p* = 0.0006, q = 0.0009), BMI (*p* < 0.001, q < 0.001), and alcohol methylation (*p* < 0.001, q < 0.001). The AHRR difference was in the same direction but did not reach the conventional level of significance (*p* = 0.056, q = 0.070). Variance inflation factors ranged from 1.04 to 1.69, providing no indication that the estimates were distorted by multicollinearity. Age and female sex were positively associated with early-life systemic dysregulation vulnerability. Neither covariate was associated with replication/mitotic aging in this model.

### 3.4. DNAm Telomere Length and Immune-Cell Composition

**Figure 4:**
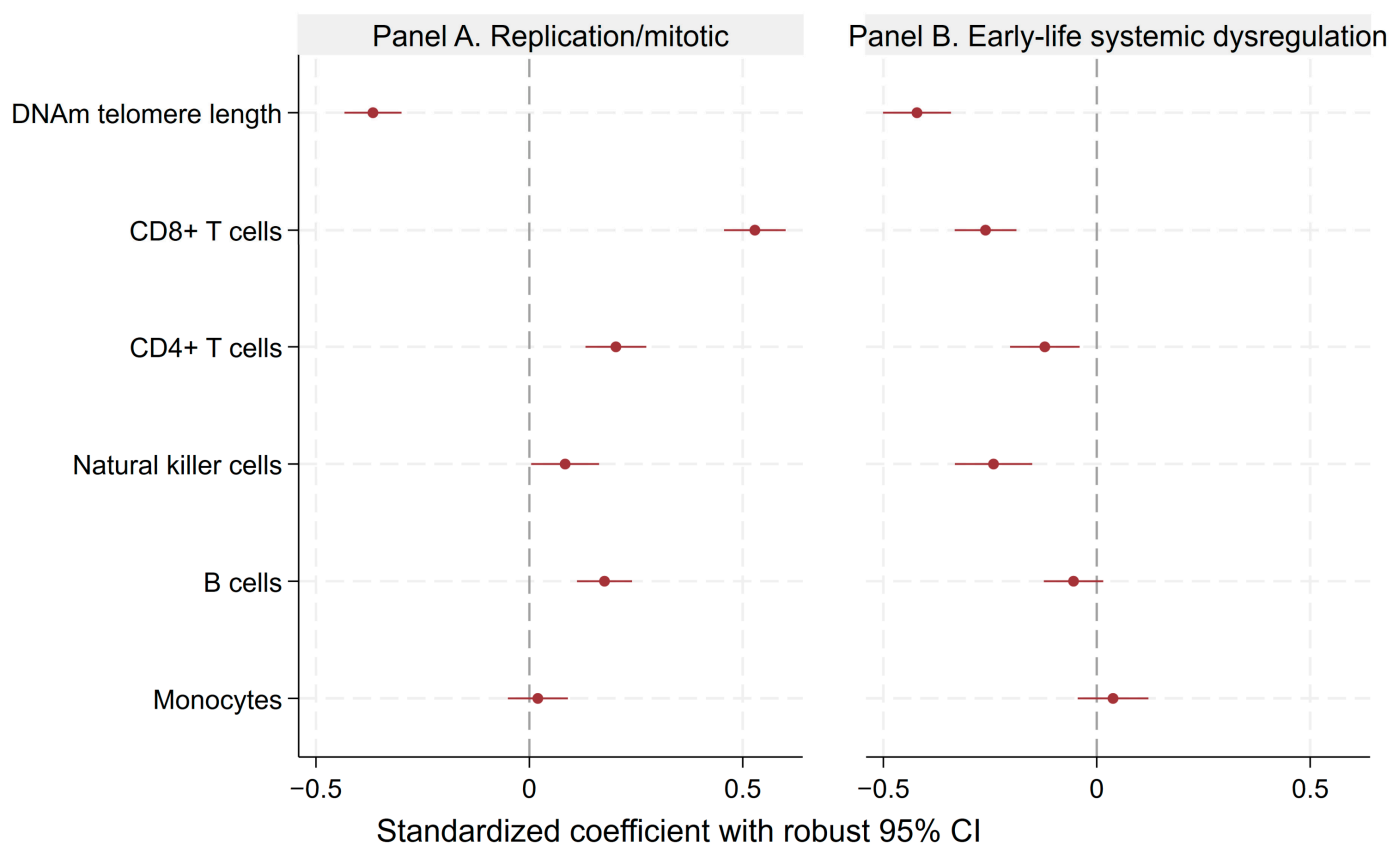
Associations of DNAm-estimated telomere length and estimated immune-cell proportions with the replication/mitotic and early-life systemic dysregulation vulnerability domains. Points are standardized regression coefficients and horizontal lines are heteroskedasticity-robust 95% confidence intervals. Each model includes DNAmTL and all five cell estimates and adjusts for mean-centered age, centered age squared, and sex. DNAmTL was inversely associated with both domains. CD8+ T cells, CD4+ T cells, natural killer cells, and B cells showed positive associations with replication/mitotic aging and negative or weaker associations with early-life systemic dysregulation vulnerability.

Table 4 and Figure 4 show the results for DNAmTL and estimated immune-cell composition. DNAmTL was negatively associated with both domains. A one-standard-deviation increase in DNAmTL was associated with a 0.366-standard-deviation decrease in replication/mitotic aging (95% CI [−0.433, −0.299], *p* < 0.001) and a 0.421-standard-deviation decrease in early-life systemic dysregulation vulnerability (95% CI [−0.501, −0.342], *p* < 0.001). The two coefficients were not significantly different (*p* = 0.247, q = 0.275). In other words, shorter methylation-estimated telomere length was related to higher scores on both domains.

The immune-cell results were different. CD8+ T cells were positively associated with replication/mitotic aging (b = 0.528, 95% CI [0.456, 0.601]) but negatively associated with early-life systemic dysregulation vulnerability (b = −0.261, 95% CI [−0.333, −0.188]). The same pattern appeared for CD4+ T cells (b = 0.203, 95% CI [0.132, 0.274] for replication/mitotic aging; b = −0.122, 95% CI [−0.203, −0.040] for early-life systemic dysregulation vulnerability) and natural killer cells (b = 0.084, 95% CI [0.004, 0.163]; b = −0.242, 95% CI [−0.332, −0.151], respectively). B cells were positively related to replication/mitotic aging (b = 0.176, 95% CI [0.112, 0.241]), but their association with early-life systemic dysregulation vulnerability was not significant. The cross-domain differences for CD8+ T cells, CD4+ T cells, natural killer cells, and B cells remained significant after false-discovery-rate adjustment (all q < 0.001).

Monocytes did not show this pattern in the simultaneous model. Their coefficients were small for replication/mitotic aging (b = 0.020, 95% CI [−0.051, 0.090]) and early-life systemic dysregulation vulnerability (b = 0.038, 95% CI [−0.045, 0.121]), and the cross-domain test was not significant (*p* = 0.704, q = 0.704). Taken together, DNAmTL and the five cell estimates explained 61.8% of the variance in replication/mitotic aging and 46.3% of the variance in early-life systemic dysregulation vulnerability. The VIFs ranged from 1.03 to 1.42. Age and female sex were positively associated with early-life systemic dysregulation vulnerability. Female sex was positively associated with replication/mitotic aging in this model, whereas the linear and quadratic age terms were not.

### 3.5. CFA Factor-Score and Selection-Weighted Sensitivity Analyses

The CFA scores were very similar to the equal-weight composites, with correlations of 0.972 for replication/mitotic aging and 0.978 for early-life systemic dysregulation vulnerability. Analyses using the factor scores gave the same overall pattern as the primary models (Table A3). CRP (b = 0.189, 95% CI [0.114, 0.265]), BMI (b = 0.283, 95% CI [0.199, 0.367]), AHRR/cg05575921 (b = 0.169, 95% CI [0.078, 0.260]), and alcohol methylation (b = 0.468, 95% CI [0.374, 0.562]) were positively associated with the early-life systemic dysregulation factor score, whereas none was associated with the replication/mitotic factor score. Cross-factor differences were significant for CRP (*p* = 0.0003), BMI (*p* < 0.001), AHRR/cg05575921 (*p* = 0.0065), and alcohol methylation (*p* < 0.001). The AHRR contrast was stronger than in the equal-weight analysis. It should still be read with the component results, which show opposite AHRR associations with PhenoAge and GrimAgeV2.

The factor-score cell models also reproduced the primary findings. DNAmTL was inversely associated with the replication/mitotic factor (b = −0.373) and the early-life systemic dysregulation factor (b = −0.377), and the coefficients did not differ (*p* = 0.935). CD8+ T cells, CD4+ T cells, natural killer cells, and B cells showed positive coefficients for the replication/mitotic factor and negative or weaker coefficients for the early-life systemic dysregulation factor. The cross-factor differences remained significant. Monocytes were unrelated to either factor score and did not differ across factors (*p* = 0.411).

The selection-weighted analyses led to the same general conclusion. In the pweighted exposure model, CRP (b = 0.161), BMI (b = 0.264), AHRR/cg05575921 (b = 0.133), and alcohol methylation (b = 0.518) remained positively associated with early-life systemic dysregulation vulnerability. The corresponding replication/mitotic coefficients remained small and nonsignificant. In the pweighted cell model, DNAmTL remained inversely associated with both domains. CD8+ T cells and CD4+ T cells remained positively associated with replication/mitotic aging and inversely associated with early-life systemic dysregulation vulnerability. Natural killer cells remained inversely associated with early-life systemic dysregulation vulnerability. Their coefficient for replication/mitotic aging did not change. The point estimate was 0.084 in both the unweighted and the weighted model, whereas the robust standard error rose from 0.040 to 0.050. That is, weighting altered the precision of this estimate rather than its magnitude (*p* = 0.098). B cells remained positively associated with replication/mitotic aging but showed no association with early-life systemic dysregulation vulnerability. Monocytes were not associated with either domain.

The factor-score models show that the findings did not depend on assigning equal weights to the component scores. The weighted models show that adjustment for the observed baseline differences did not remove the pattern. Neither analysis addresses the MiAge boundary condition, selection on unmeasured characteristics, or the common methylation source of several measures.

### 3.6. Component-Level and Other Sensitivity Analyses

We then ran the same model separately for CellDRIFT, EpiTOC2, and MiAge (Table A4). None of the four predictors reached *p* < 0.05 for any of the three scores. The largest coefficient was for CRP and CellDRIFT (b = −0.095, *p* = 0.085). The null result for the replication/mitotic domain was therefore not due to opposite associations across the three component scores.

The component results for early-life systemic dysregulation vulnerability were more varied. BMI was positively associated with PhenoAge (b = 0.162, *p* = 0.001), GrimAgeV2 (b = 0.179, *p* < 0.001), and DunedinPACE (b = 0.307, *p* < 0.001). Alcohol methylation also had positive coefficients for all three measures (b = 0.477 for PhenoAge, 0.442 for GrimAgeV2, and 0.357 for DunedinPACE; all *p* < 0.001). CRP was associated with GrimAgeV2 (b = 0.132, *p* < 0.001) and DunedinPACE (b = 0.211, *p* < 0.001), but not with PhenoAge. AHRR did not show a consistent pattern. The results showed that it was negatively associated with PhenoAge (b = −0.211, *p* < 0.001), positively associated with GrimAgeV2 (b = 0.405, *p* < 0.001), and not related to DunedinPACE. The strong association between the AHRR/cg05575921 smoking measure and GrimAgeV2 is expected in part by construction, because GrimAgeV2 incorporates a DNAm surrogate for smoking; this coefficient should therefore not be read as independent confirmation of a smoking–aging association.

The leave-one-score-out analyses are shown in Table A5. CRP, BMI, and alcohol methylation remained positively associated with the early-life systemic dysregulation composite no matter which score was removed. The AHRR result was less stable. After removing GrimAgeV2, the coefficient became negative and was not significant (b = −0.071, *p* = 0.149). After removing PhenoAge, it was positive and much larger (b = 0.272, *p* < 0.001). After removing DunedinPACE, it remained positive but was smaller (b = 0.114, *p* = 0.017). These results show that the positive AHRR coefficient in the primary composite was driven mainly by GrimAgeV2 rather than being common across all three early-life systemic dysregulation measures.

We also removed linear and quadratic age from each DNAm score and repeated the structural analyses. The result changed very little. In the age-residualized PCA, Component 1 explained 45.3% of the variance and Component 2 explained 32.1%; together they explained 77.4%. The age-residualized correlations closely resembled those in Table 1. The separation of the two groups was therefore not produced simply by differences in chronological age.

We estimated separate models for each immune-cell proportion (Table A6). When the cell estimates were entered one at a time with DNAmTL and the covariates, CD8+ T cells, CD4+ T cells, natural killer cells, and B cells remained positively associated with replication/mitotic aging and negatively associated with early-life systemic dysregulation vulnerability. But the monocyte result was mixed. When the other cell estimates were omitted, monocytes were positively associated with replication/mitotic aging (b = 0.185, *p* < 0.001) and inversely associated with early-life systemic dysregulation vulnerability (b = −0.094, *p* = 0.031). This change suggests that the null monocyte coefficients in the simultaneous model reflect adjustment for the other estimated cell fractions. The single-cell models support the broad contrast between the two domains, but they do not remove the compositional dependence among blood-cell estimates. For the cell-composition analyses, we also estimated separate models for each cell fraction together with DNAmTL and the covariates. These models do not remove the sum-to-one constraint because a higher proportion of one cell type still means a lower proportion of one or more other cell types. We used these models only to determine whether the direction of an association changed when the other cell estimates were not included.

**Figure A1:**
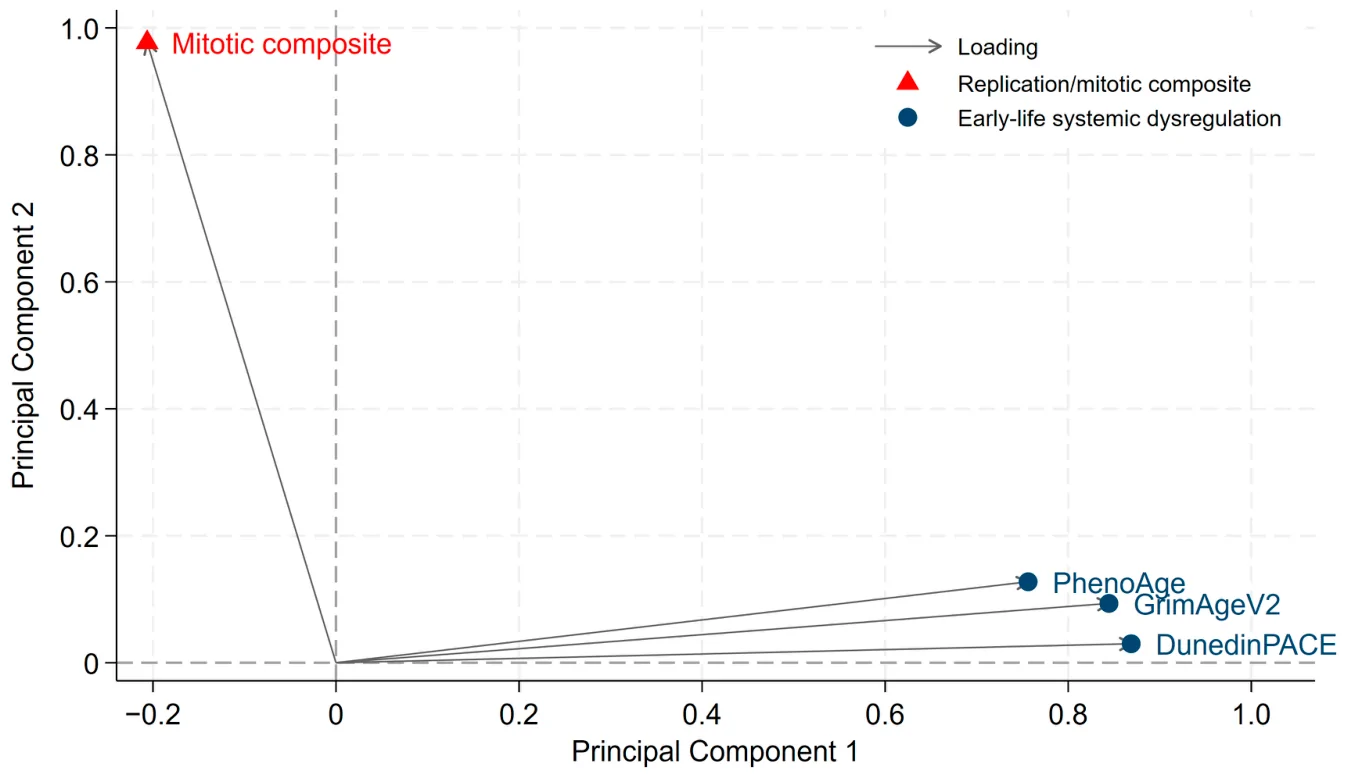
Principal component loading plot of the mitotic composite and three early-life systemic dysregulation measures.

Finally, because of the high coherence among the three replication/mitotic scores, we repeated the structural analysis using their standardized equal-weight composite as a single measure. The mitotic composite was not correlated with PhenoAge, GrimAgeV2, and DunedinPACE (see Table A7). In the four-variable PCA, PhenoAge, GrimAgeV2, and DunedinPACE loaded primarily on the first component, whereas the mitotic composite loaded primarily on the second component. Thus, the separation remained when CellDRIFT, EpiTOC2, and MiAge were represented by a single composite rather than three separate scores (see Figure A1).

## 4. Discussion

Although recent reviews have focused on the development of first-, second-, and third-generation epigenetic clocks, replication/mitotic methylation clocks remain less integrated into population-based epigenetic aging research. This gap is important because mitotic clocks may capture hematopoietic cell-turnover and replication-linked methylation biology that is distinct from the early-life systemic dysregulation captured by GrimAgeV2, PhenoAge, and DunedinPACE.

Our evaluation of blood-based DNAm scores revealed a distinct biological bifurcation between mitotic/replicative aging and early-life systemic dysregulation vulnerability. Rather than showing a singular underlying axis of biological age, these epigenetic clocks naturally clustered into two groups of domains that reflect fundamentally different aspects of aging biology. The first domain—comprising clocks that emphasize cellular division (CellDRIFT, EpiTOC2, and MiAge)—tracks cellular turnover and immune system dynamics, and in this sample the three scores were close to interchangeable. It was positively associated with the estimated proportions of CD8+ T, CD4+ T, natural killer, and B cells, whereas the early-life systemic dysregulation domain showed negative or null associations with the same estimates. Our findings align with evidence that methylation-based clocks count cell-specific division and are highly sensitive to shifts in blood cell subtypes. For example, EpiTOC reflects cumulative cell division by assessing methylation increases in polycomb group (PcG) target promoters [1]. Since these scores are closely associated with white blood cell composition and replication history, they appear to capture hematopoietic lineage expansion and replicative history, rather than overall physiological wear-and-tear.

In contrast, the second domain—encompassing phenotypic and mortality clocks (PhenoAge, GrimAgeV2, and DunedinPACE)—serves as an indicator of early-life systemic dysregulation strain. Cardiometabolic risk factors and exposures, including BMI, CRP, alcohol consumption, and smoking (AHRR/cg05575921), show strong associations with this early-life systemic dysregulation domain while remaining largely unlinked to mitotic scores. These physiological clocks consistently show early-life systemic dysregulation in socially disadvantaged populations, functioning as dynamic sensors of structural adversity [43,44]. The two domains were not simply unrelated. The composite scores correlated −0.108 and the latent factors correlated −0.114, and the same pattern held after linear and quadratic age were removed from each score. A negative correlation is not what a single aging process would produce. The cell-composition results point to one explanation. Higher estimated proportions of CD8+ T, CD4+ T, and natural killer cells were associated with higher replication/mitotic scores and lower early-life systemic dysregulation scores, so any influence that shifts leukocyte composition moves the two domains in opposite directions. That is, the domains may be separated by blood-cell composition rather than two aging processes running at different rates. The correlation is small and accounts for roughly one percent of shared variance, so this pattern constrains interpretation more than it establishes a mechanism.

Interestingly, DNAmTL blurred this domain boundary, showing inverse associations with both mitotic and early-life systemic dysregulation metrics. DNAmTL reflects cell replication history more directly than actual telomere length, providing a mechanistic link between cellular replication and age-related pathologies [1,45]. Furthermore, DNAmTL exhibits moderate inverse correlations with age acceleration measures from both PhenoAge and GrimAge, suggesting that it operates distinctly from standard epigenetic clocks [45]. This suggests that while epigenetic clocks capture different biological processes, telomere dynamics may represent an overarching constraint shared by both replicative capacity and general physiological decline.

This distinction between two domains is especially important for social determinants of health research. Social disadvantages, discrimination, chronic stress, and unequal exposure to harmful environments are thought to become biologically embedded through inflammatory, metabolic, neuroendocrine, and immune pathways [46,47,48]. Most population-based epigenetic aging studies have examined this process using general aging clocks such as PhenoAge, GrimAge, and DunedinPACE, which are closely tied to morbidity, mortality, smoking, inflammation, and physiological dysregulation rather than replication-linked methylation biology [49,50]. In this context, CRP, BMI, alcohol methylation, and AHRR/cg05575921 are better interpreted not simply as isolated risk factors but as correlates of a broader early-life systemic dysregulation vulnerability domain through which social conditions may become biologically embedded [50,51,52].

If replication/mitotic methylation clocks capture hematopoietic cell turnover and replication-linked methylation biology, they may identify an additional pathway through which social conditions influence cancer disparities. This matters because cancer risk is not driven by a single aging process. It reflects the combined effects of socially patterned exposures, immune function, chronic inflammation, environmental stressors, and differential access to health-promoting resources and care [46,47,53]. Mitotic age is especially relevant in this context because cumulative stem-cell divisions and increased tissue turnover are thought to contribute directly to neoplastic transformation, and DNAm-based mitotic clocks are accelerated in precancerous lesions, cancer, and normal tissues exposed to major carcinogenic risk factors [9,12,29]. Distinguishing early-life systemic dysregulation vulnerability from replication/mitotic methylation biology therefore allows cancer disparities research to move beyond the broad claim that adversity “accelerates aging” and toward a more specific model of whether social conditions act primarily through systemic early-life systemic dysregulation, replication-linked tissue turnover, or both [46,52].

This study has several limitations that we would like to acknowledge. First, it did not include a direct cancer endpoint, so the findings establish domain structure rather than cancer risk prediction or cancer causation. Second, our sample was relatively young and modest in size. Participants were approximately 28.7 years old at Wave 7, so the term “early-life systemic dysregulation” should be interpreted as aging-related biological vulnerability rather than late-life aging. In addition, the final sample included 420 participants from the larger longitudinal cohort. Because the analysis was limited to participants with methylation and complete data, the findings may not fully represent the original cohort. Third, the estimated immune-cell fractions are compositional. Because the full set of cell fractions sums to one, the coefficients should not be interpreted as the independent effects of individual cell types. The single-cell models also do not remove this constraint. In addition, several cell types were positively associated with the replication/mitotic domain but negatively associated with the early-life systemic dysregulation domain. Therefore, differences in blood-cell composition may partly explain the modest negative correlation between the two domains. Fourth, AHRR/cg05575921 and the Alcohol T Score were measured separately using MSdPCR and were not calculated from the EPIC array. However, they are still DNA methylation measures. Their associations with the EPIC-derived aging measures may partly reflect common methylation signals. This is especially important for AHRR/cg05575921 because GrimAgeV2 includes a DNAm-based measure of smoking. Serum CRP and measured BMI, which were obtained independently of DNAm, provide more independent phenotypic tests of the early-life systemic dysregulation domain. Finally, the use of a single DNAm assessment prevents conclusions about whether these domains reflect longitudinal change, cumulative exposure, or stable individual differences. In addition, our study did not include cancer incidence, mortality, or family-history measures. Therefore, we cannot determine whether either domain predicts cancer risk.

Future research should prioritize large-scale, longitudinal study designs to track methylation dynamics over time and validate whether epigenetic aging domains predict actual cancer incidence [19,22,54]. Since current clocks often rely on linear models, new computational approaches are needed to detect non-linear aging trajectories and sex-specific transitions that may better capture early disease risk [55,56]. The most notable gap is the lack of multi-omics interpretation in socially disadvantaged populations, despite strong evidence that diverse cohorts are needed to generalize epigenetic findings [43,54]. While multi-omics is actively pursued in general aging research [57], applying these integrated models to social determinants and cancer disparities remains largely unexplored.

## 5. Conclusions

These findings suggest that blood-based DNAm aging measures do not reflect a single aging process. Instead, they distinguish at least two separable methylation domains: one reflecting replication-driven mitotic aging and hematopoietic cell-turnover biology, and another reflecting early-life systemic dysregulation vulnerability linked to inflammation, metabolic risk, smoking methylation, and alcohol-related methylation. Because cancer outcomes were not examined, the cancer relevance of these domains is mechanistic and inferential. They should not be interpreted as tumor-derived biomarkers or validated cancer predictor scores. This two-domain framework may help clarify how blood DNAm profiles capture complementary biological processes relevant to cancer susceptibility and guide future studies of methylation-based cancer risk. Distinguishing these domains also gives social determinants research a more specific way to ask how social conditions may contribute to cancer risk: through systemic early-life systemic dysregulation, through replication-linked tissue turnover, or through both.

## Figures and Tables

**Table 1:** Bivariate correlations among the six DNAm scores.

Variables	(1)	(2)	(3)	(4)	(5)	(6)
(1) CellDRIFT	1.000					
(2) EpiTOC2	0.711 **	1.000				
(3) MiAge	0.863 **	0.842 **	1.000			
(4) PhenoAge	−0.099 *	−0.037	−0.033	1.000		
(5) GrimAgeV2	−0.076	−0.066	−0.082	0.449 **	1.000	
(6) DunedinPACE	−0.131 **	−0.118 *	−0.105 *	0.492 **	0.643 **	1.000

Note. *N* = 420. ** *p* < 0.01; * *p* < 0.05 (two-tailed tests).

**Table 2:** Confirmatory factor analysis of the six DNAm scores.

Model	χ (df)	CFI	TLI	RMSEA	SRMR	ΔAIC	ΔBIC
One factor	370.38 (10)	0.752	0.628	0.293	0.201	351.0	347.0
Correlated two factor	17.35 (9)	0.994	0.990	0.047	0.019	0.0	0.0

Note. *N* = 420. The one-factor model *p* value was <0.001; the correlated two-factor model *p* value was 0.044. Delta AIC and Delta BIC are differences relative to the two-factor model; lower values indicate better fit. Both reported models fixed the MiAge residual variance at 0.01 because the unconstrained one- and two-factor solutions reached the zero boundary.

**Table 3:** Exposure and inflammatory predictors of the two DNAm domains.

Predictor	Replication/Mitotic Domain b [95% CI]	Early-Life Systemic Dysregulation Vulnerability b [95% CI]	Cross-Domain *p*	BH-FDR q
CRP	−0.077 [−0.188, 0.033]	0.164 [0.086, 0.242]	0.0006	0.0009
BMI	−0.080 [−0.201, 0.042]	0.261 [0.178, 0.345]	<0.001	<0.001
AHRR/cg05575921	−0.041 [−0.170, 0.088]	0.114 [0.022, 0.205]	0.056	0.070
Alcohol methylation	−0.007 [−0.145, 0.131]	0.514 [0.421, 0.607]	<0.001	<0.001
R-square	0.017	0.478		

Note. *N* = 420. Entries are standardized regression coefficients with heteroskedasticity-robust 95% confidence intervals. Both models include CRP, BMI, AHRR/cg05575921, and alcohol methylation and adjust for mean-centered age, centered age squared, and sex. Cross-domain *p* values are based on seemingly unrelated estimation. BH-FDR q values adjust the 10 cross-domain tests.

**Table 4:** DNAm telomere length and immune-cell predictors of the two DNAm domains.

Predictor	Replication/Mitotic Domain b [95% CI]	Early-Life Systemic Dysregulation Vulnerability b [95% CI]	Cross-Domain *p*	BH-FDR q
DNAm telomere length	−0.366 [−0.433, −0.299]	−0.421 [−0.501, −0.342]	0.247	0.275
CD8+ T cells	0.528 [0.456, 0.601]	−0.261 [−0.333, −0.188]	<0.001	<0.001
CD4+ T cells	0.203 [0.132, 0.274]	−0.122 [−0.203, −0.040]	<0.001	<0.001
Natural killer cells	0.084 [0.004, 0.163]	−0.242 [−0.332, −0.151]	<0.001	<0.001
B cells	0.176 [0.112, 0.241]	−0.054 [−0.124, 0.015]	<0.001	<0.001
Monocytes	0.020 [−0.051, 0.090]	0.038 [−0.045, 0.121]	0.704	0.704
Model R2	0.618	0.463		

Note. *N* = 420. Entries are standardized regression coefficients with heteroskedasticity-robust 95% confidence intervals. Both models include DNAmTL and all five estimated immune-cell proportions and adjust for mean-centered age, centered age squared, and sex. Cross-domain *p* values are based on seemingly unrelated estimation. BH-FDR q values adjust the 10 cross-domain tests.

## Data Availability

Although the data are not publicly available due to potential problems with identifiability, the analytic code is available in Appendix B.

## Abbreviations

The following abbreviations are used in this manuscript:

DNAmTL	DNA methylation-based estimator of telomere length
AHRR	aryl hydrocarbon receptor repressor
CpG	cytosine–phosphate–guanine
CRP	C-reactive protein
DNAm	DNA methylation
DNMT	DNA methyltransferase
VIF	Variance inflation factors

## Appendix A

Whole blood samples were assessed for genome-wide methylation levels using standard protocols. The phlebotomist drew four tubes of blood (30 mL) from each participant, which were shipped on the same day to a laboratory at the University of Iowa for preparation. Whole blood DNA was prepared using cold protein precipitation, quantified with a NanoDrop spectrophotometer (Thermo Fisher Scientific, Waltham, MA, USA), and stored at −20 °C until use [27]. The Illumina Infinium HumanMethylationEPIC BeadChip (850K; EPIC) was used to assay genome-wide DNA methylation at the University of Minnesota Genome Center according to the manufacturer’s instructions (Illumina, San Diego, CA, USA). This array contains 865,918 probes targeting CpG positions associated with known transcripts, potential transcripts, or CpG islands.

Samples were randomly assigned across 16 BeadChips, with groups of eight BeadChips being bisulfite converted on a single plate, resulting in two “batches/plates.” A replicate DNA sample was included on each plate to assess batch variation and ensure correct handling of specimens. The replicate sample was examined for the correlation of beta values between plates, which was greater than 0.99. Prior to normalization, DNA methylation data were filtered based the following criteria: (a) samples were examined to identify any “poor quality samples” with 1% or more of CpG sites with detection *p* < 0.05 (no samples failed this criterion); (b) sites were removed if a bead count of <3 was present in 5% of samples; and (c) sites with a detection *p* < 0.05 in 1% of samples were removed. The resulting data were normalized using the DASEN method with the MethyLumi, WateRmelon and IlluminaHumanMethylationEPICanno.ilm10b2.hg19 R packages. CpG values were background-corrected using the NOOB method. The beta value at each CpG locus was calculated as the ratio of the intensity of the methylated probe to the sum of the intensities of the methylated and unmethylated probes. Finally, the data were subjected to standard sample- and probe-level quality control measures. Details regarding the preparation of the methylation data are described by Beach and colleagues [58].

**Table A1:** Analytic sample construction.

Step	*N* Retained	*N* Excluded at Step	Reason for Exclusion
FACHS baseline target sample	889		Original recruited target families
Wave 7 valid EPIC methylation sample	449	440	No valid Wave 7 methylation/blood analytic record
Complete focal exposure/inflammation data	420	29	Missing at least one of AHRR/cg05575921, alcohol methylation, CRP, or BMI
Complete age, sex, DNAmTL, and cell data	420	0	No additional exclusions

Note. FACHS began with 889 target participants. At Wave 7, 449 had valid EPIC-array methylation data. The 440-person difference reflects longitudinal nonparticipation, lack of blood collection, absence of a valid methylation result, or some combination of these processes; the available files do not separate them. Within the 449-person methylation sample, 29 participants were missing at least one focal exposure or inflammatory measure. Three did not match the CRP file, and the other 26 had a missing value on at least one focal measure after merging. No further case was excluded because of missing DNAm scores, age, sex, DNAmTL, or immune-cell estimates. The final analytic sample was 420.

**Table A2:** Baseline characteristics of participants included and not included in the analytic sample.

Baseline Characteristic	Included (*N* = 420)	Not Included (*N* = 469)	SMD	*p* Value
Age, years	10.95 (0.61)	11.07 (0.58)	−0.20	0.003
Male, %	37.6	53.9	−0.33	<0.001
Iowa, %	39.8	64.0	−0.48	<0.001
Household poverty, %	56.1	59.0	−0.06	0.446
Parent education below high school, %	29.8	32.0	−0.05	0.474
Single-parent family, %	50.2	44.8	0.11	0.103
Missing baseline poverty, %	25.2	25.2	0.00	0.979

Note. Values are means (standard deviations) or percentages. SMD = standardized mean difference, calculated as included minus not included. The family-structure comparison using all six original categories was also not significant (chi-square [df = 5] = 8.45, *p* = 0.133). Household poverty information was available for 665 participants. Baseline age was missing for one participant in the not-included group, so the age comparison is based on 888 participants (420 included, 468 not included).

**Table A3:** CFA factor-score sensitivity analyses.

Predictor	Replication/Mitotic Factor b [95% CI]	Early-Life Systemic Dysregulation b [95% CI]	Cross-Factor *p*
CRP	−0.060 [−0.166, 0.047]	0.189 [0.114, 0.265]	0.0003
BMI	−0.068 [−0.186, 0.050]	0.283 [0.199, 0.367]	<0.001
AHRR/cg05575921	−0.054 [−0.184, 0.075]	0.169 [0.078, 0.260]	0.0065
Alcohol methylation	0.005 [−0.131, 0.142]	0.468 [0.374, 0.562]	<0.001
DNAm telomere length	−0.373 [−0.440, −0.306]	−0.377 [−0.458, −0.297]	0.935
CD8+ T cells	0.537 [0.468, 0.605]	−0.261 [−0.335, −0.187]	<0.001
CD4+ T cells	0.214 [0.142, 0.286]	−0.126 [−0.208, −0.044]	<0.001
Natural killer cells	0.075 [−0.001, 0.151]	−0.267 [−0.356, −0.179]	<0.001
B cells	0.142 [0.076, 0.209]	−0.018 [−0.089, 0.054]	0.0006
Monocytes	0.003 [−0.068, 0.073]	0.045 [−0.040, 0.129]	0.411

Note. *N* = 420. Factor scores were generated from the correlated two-factor CFA after fixing the MiAge residual variance at 0.01 to avoid the boundary estimate in the unconstrained model. Factor scores and continuous predictors were standardized. Models use heteroskedasticity-robust standard errors and adjust for mean-centered age, centered age squared, and sex.

**Table A4:** Component-level exposure and inflammatory models.

DNAm Score	CRP	BMI	AHRR/cg05575921	Alcohol Methylation	R2
CellDRIFT	−0.095	−0.061	−0.042	−0.031	0.032
EpiTOC2	−0.063	−0.096	−0.017	0.005	0.030
MiAge	−0.058	−0.066	−0.055	0.007	0.012
PhenoAge	0.064	0.162 **	−0.211 **	0.477 **	0.234
GrimAgeV2	0.132 **	0.179 **	0.405 **	0.442 **	0.605
DunedinPACE	0.211 **	0.307 **	0.088	0.357 **	0.377

Note. *N* = 420. Entries are standardized coefficients from separate robust linear regression models for each DNAm score. Each equation includes CRP, BMI, AHRR/cg05575921, alcohol methylation, mean-centered age, centered age squared, and sex. ** *p* < 0.01. The CRP coefficient for CellDRIFT (*p* = 0.085) and the AHRR coefficient for DunedinPACE (*p* = 0.077) are not marked.

**Table A5:** Leave-one-score-out early-life systemic dysregulation vulnerability models.

Early-Life Systemic Dysregulation Composite	CRP	BMI	AHRR/cg05575921	Alcohol Methylation	R2
Without GrimAgeV2	0.159 **	0.272 **	−0.071	0.483 **	0.373
Without PhenoAge	0.189 **	0.268 **	0.272 **	0.441 **	0.521
Without DunedinPACE	0.115 **	0.201 **	0.114 *	0.540 **	0.462

Note. *N* = 420. Each composite was formed by averaging the two early-life systemic dysregulation scores remaining after the score named in the first column was omitted; the resulting mean was then standardized. Entries are standardized coefficients from robust linear regressions that include all four focal predictors and adjust for mean-centered age, centered age squared, and sex. ** *p* < 0.01; * *p* < 0.05.

**Table A6:** Single-cell sensitivity models.

Estimated Cell Proportion	Replication/Mitotic Domain b	Early-Life Systemic Dysregulation Vulnerability b
CD8+ T cells	0.667 **	−0.359 **
CD4+ T cells	0.501 **	−0.300 **
Natural killer cells	0.285 **	−0.328 **
B cells	0.400 **	−0.192 **
Monocytes	0.185 **	−0.094 *

Note. *N* = 420. Each row comes from a separate robust linear regression including one estimated cell proportion, DNAmTL, mean-centered age, centered age squared, and sex. ** *p* < 0.01; * *p* < 0.05. Because estimated blood-cell fractions are compositional, these models do not identify effects of isolated cell populations.

**Table A7:** Mitotic composite and early-life systemic dysregulation scores.

Variables	(1)	(2)	(3)	(4)
(1) Standardized mitotic	1.000			
(2) Standardized PhenoAge	−0.061	1.000		
(3) Standardized GrimAgeV2	−0.080	0.449 **	1.000	
(4) Standardized DunedinPACE	−0.126 **	0.492 **	0.643 **	1.000

** *p* < 0.01.

## Appendix B. Analytic Code Used in Data Analysis

/**********************************************************************

Cancer-Relevant DNA Methylation Domains

**********************************************************************/

* *

clear all

set more off

version 19

* *

cd "."

* *

capture mkdir "results"

capture mkdir "figures"

* *

capture log close

log using "results\cancer_domains_simple_check.log", replace text

* *

/**********************************************************************

1. BASELINE SAMPLE-SELECTION CHECK

**********************************************************************/

* *

use "cancer_domains_analytic_corrected.dta", clear

isid caseid

count

assert r(N) == 420

* *

preserve

keep caseid

gen byte included = 1

save "_included_ids_for_simple_check.dta", replace

restore

* *

use "Baseline_targets.dta", clear

isid caseid

count

assert r(N) == 889

* *

merge 1:1 caseid using "_included_ids_for_simple_check.dta", keep(master match) nogen

replace included = 0 if missing(included)

tabulate included

* *

* Included versus not included.

ttest AgeYW1d, by(included) unequal

* *

tabulate male included, row chi2

tabulate Iowa included, row chi2

tabulate poverty1 included if !missing(poverty1), row chi2

tabulate LessHS1 included, row chi2

* *

gen byte single_parent = inlist(Fstruct1,3,4)

tabulate single_parent included, row chi2

tabulate Fstruct1 included, row chi2

* *

gen byte poverty_missing = missing(poverty1)

tabulate poverty_missing included, row chi2

* *

* Selection model and stabilized selection weights.

summarize poverty1

gen double poverty_imp = poverty1

replace poverty_imp = r(mean) if missing(poverty_imp)

* *

summarize AgeYW1d

gen double age1_imp = AgeYW1d

replace age1_imp = r(mean) if missing(age1_imp)

* *

logit included c.age1_imp c.poverty_imp i.poverty_missing ///

  i.LessHS1 i.male i.Iowa i.Fstruct1, vce(robust)

* *

predict double p_select, pr

gen double sw_select = (420/889)/p_select if included == 1

summarize sw_select, detail

* *

keep if included == 1

keep caseid sw_select p_select

save "_selection_weights_for_simple_check.dta", replace

* *

/**********************************************************************

2. PREPARE THE 420-PERSON ANALYTIC DATASET

**********************************************************************/

* *

use "cancer_domains_analytic_corrected.dta", clear

merge 1:1 caseid using "_selection_weights_for_simple_check.dta", assert(3) nogen

* *

capture drop age_c age_c2 AHRR_smoking

capture drop z_CellDRIFTW7 z_epiTOC2W7 z_MiAgeW7

capture drop z_PhenoAgeW7 z_GrimAgeV2W7 z_DunedinPACETT7

capture drop z_AHRR_smoking z_ATS z_CRP z_BMI

capture drop z_DNAmTLW7 z_CD8T_W7 z_CD4T_W7 z_NK_W7 z_Bcell_W7 z_Mono_W7

capture drop mito_raw phys_raw z_mitotic_domain z_physaging_domain

capture drop fs_mitotic fs_physio z_fs_mitotic z_fs_physio

capture drop phys_no_grim_raw z_phys_no_grim

capture drop phys_no_pheno_raw z_phys_no_pheno

capture drop phys_no_pace_raw z_phys_no_pace

capture drop r_CellDRIFT r_epiTOC2 r_MiAge r_PhenoAge r_GrimAgeV2 r_DunedinPACE

capture drop zr_CellDRIFT zr_epiTOC2 zr_MiAge zr_PhenoAge zr_GrimAgeV2 zr_DunedinPACE

* *

summarize AgeW7

gen double age_c = AgeW7 - r(mean)

gen double age_c2 = age_c^2

* *

* Six DNAm scores.

egen z_CellDRIFTW7 = std(CellDRIFTW7)

egen z_epiTOC2W7 = std(epiTOC2W7)

egen z_MiAgeW7 = std(MiAgeW7)

egen z_PhenoAgeW7 = std(PhenoAgeW7)

egen z_GrimAgeV2W7 = std(GrimAgeV2W7)

egen z_DunedinPACETT7 = std(DunedinPACETT7)

* *

* Exposure and inflammatory measures.

gen double AHRR_smoking = -1*SS

egen z_AHRR_smoking = std(AHRR_smoking)

egen z_ATS = std(WBATS)

egen z_CRP = std(CRPTT7)

egen z_BMI = std(BMI7TTTO)

* *

* DNAm telomere length and estimated immune cells.

egen z_DNAmTLW7 = std(DNAmTLW7)

egen z_CD8T_W7 = std(CD8T_W7)

egen z_CD4T_W7 = std(CD4T_W7)

egen z_NK_W7 = std(NK_W7)

egen z_Bcell_W7 = std(Bcell_W7)

egen z_Mono_W7 = std(Mono_W7)

* *

label variable z_CRP "CRP"

label variable z_BMI "BMI"

label variable z_AHRR_smoking "AHRR/cg05575921"

label variable z_ATS "Alcohol methylation"

label variable z_DNAmTLW7 "DNAm telomere length"

label variable z_CD8T_W7 "CD8+ T cells"

label variable z_CD4T_W7 "CD4+ T cells"

label variable z_NK_W7 "Natural killer cells"

label variable z_Bcell_W7 "B cells"

label variable z_Mono_W7 "Monocytes"

* *

summarize AgeW7 FemaleW7 CellDRIFTW7 epiTOC2W7 MiAgeW7 ///

  PhenoAgeW7 GrimAgeV2W7 DunedinPACETT7 ///

  CRPTT7 BMI7TTTO SS WBATS DNAmTLW7 ///

  CD8T_W7 CD4T_W7 NK_W7 Bcell_W7 Mono_W7

* *

/**********************************************************************

3. FIGURE 1, CORRELATIONS, AND PCA

* *

MDPI figure-format note:

Axis tick labels are specified manually so that negative values use the

Unicode minus sign (U+2212) and decimals include a leading zero.

**********************************************************************/

* *

twoway ///

  (kdensity z_CellDRIFTW7, lwidth(medthick)) ///

  (kdensity z_epiTOC2W7, lwidth(medthick)) ///

  (kdensity z_MiAgeW7, lwidth(medthick)) ///

  (kdensity z_PhenoAgeW7, lwidth(medthick)) ///

  (kdensity z_GrimAgeV2W7, lwidth(medthick)) ///

  (kdensity z_DunedinPACETT7, lwidth(medthick)), ///

  legend(order(1 "CellDRIFT" 2 "EpiTOC2" 3 "MiAge" ///

        4 "PhenoAge" 5 "GrimAgeV2" 6 "DunedinPACE") ///

      rows(2) position(6) size(small)) ///

  xtitle("Standardized DNAm score") ///

  ytitle("Density") ///

  xlabel(-4 "−4" -2 "−2" 0 "0" 2 "2" 4 "4") ///

  ylabel(0 "0" 0.1 "0.1" 0.2 "0.2" 0.3 "0.3" 0.4 "0.4") ///

  graphregion(color(white)) plotregion(color(white))

* *

graph export "figures\Figure1_Density_simple_check.png", replace width(4000)

* *

pwcorr z_CellDRIFTW7 z_epiTOC2W7 z_MiAgeW7 ///

  z_PhenoAgeW7 z_GrimAgeV2W7 z_DunedinPACETT7, sig obs

* *

pca z_CellDRIFTW7 z_epiTOC2W7 z_MiAgeW7 ///

  z_PhenoAgeW7 z_GrimAgeV2W7 z_DunedinPACETT7

* *

matrix V = e(L)

matrix EV = e(Ev)

matrix LOAD = V[1...,1..2]

* *

matrix LOAD[1] = LOAD[1]*sqrt(EV[1])

matrix LOAD[1,2] = LOAD[1,2]*sqrt(EV[1])

matrix LOAD[1,3] = LOAD[1,3]*sqrt(EV[1])

matrix LOAD[1,4] = LOAD[1,4]*sqrt(EV[1])

matrix LOAD[1,5] = LOAD[1,5]*sqrt(EV[1])

matrix LOAD[1,6] = LOAD[1,6]*sqrt(EV[1])

* *

matrix LOAD[1,2] = LOAD[1,2]*sqrt(EV[1,2])

matrix LOAD[2] = LOAD[2]*sqrt(EV[1,2])

matrix LOAD[2,3] = LOAD[2,3]*sqrt(EV[1,2])

matrix LOAD[2,4] = LOAD[2,4]*sqrt(EV[1,2])

matrix LOAD[2,5] = LOAD[2,5]*sqrt(EV[1,2])

matrix LOAD[2,6] = LOAD[2,6]*sqrt(EV[1,2])

* *

matrix colnames LOAD = load1 load2

matrix list LOAD

* *

preserve

clear

svmat double LOAD, names(col)

* *

gen str20 score = ""

replace score = "CellDRIFT" in 1

replace score = "EpiTOC2" in 2

replace score = "MiAge" in 3

replace score = "PhenoAge" in 4

replace score = "GrimAgeV2" in 5

replace score = "DunedinPACE" in 6

* *

gen byte domain = 1 in 1/3

replace domain = 2 in 4/6

* *

gen double x0 = 0

gen double y0 = 0

* *

gen double labx = load1

gen double laby = load2

* *

* Move labels well away from the PCA points/arrows so that none overlap.

* Replication/mitotic scores: place each label in a separate vertical band.

replace labx = load1 + .150 if score == "MiAge"

replace laby = load2 + .055 if score == "MiAge"

replace labx = load1 + .150 if score == "EpiTOC2"

replace laby = load2 + .005 if score == "EpiTOC2"

replace labx = load1 + .150 if score == "CellDRIFT"

replace laby = load2 - .065 if score == "CellDRIFT"

* *

* Early-life systemic dysregulation scores: separate labels clearly.

replace labx = load1 + .100 if score == "GrimAgeV2"

replace laby = load2 + .055 if score == "GrimAgeV2"

replace labx = load1 + .100 if score == "PhenoAge"

replace laby = load2 - .035 if score == "PhenoAge"

replace labx = load1 - .200 if score == "DunedinPACE"

replace laby = load2 + .005 if score == "DunedinPACE"

* *

twoway ///

  (pcarrow y0 x0 load2 load1, lcolor(gs6) mcolor(gs6) lwidth(thin)) ///

  (scatter load2 load1 if domain == 1, ///

    msymbol(triangle) msize(medlarge) mcolor(red)) ///

  (scatter load2 load1 if domain == 2, ///

    msymbol(circle) msize(medlarge) mcolor(navy)) ///

  (scatter laby labx if domain == 1, msymbol(none) ///

    mlabel(score) mlabpos(0) mlabsize(small) mlabcolor(red)) ///

  (scatter laby labx if domain == 2, msymbol(none) ///

    mlabel(score) mlabpos(0) mlabsize(small) mlabcolor(navy)), ///

  xline(0, lpattern(dash) lcolor(gs10)) ///

  yline(0, lpattern(dash) lcolor(gs10)) ///

  legend(order(1 "Loading" 2 "Replication/mitotic" ///

    3 "Early-life systemic dysregulation") position(2) ring(0) cols(1) ///

    size(small) region(lcolor(none))) ///

  xtitle("Principal Component 1 (45.2% explained variance)") ///

  ytitle("Principal Component 2 (32.7% explained variance)") ///

  xlabel(-0.6 "−0.6" -0.4 "−0.4" -0.2 "−0.2" 0 "0" ///

    0.2 "0.2" 0.4 "0.4" 0.6 "0.6" 0.8 "0.8" 1.0 "1.0") ///

  ylabel(0 "0" 0.2 "0.2" 0.4 "0.4" 0.6 "0.6" 0.8 "0.8") ///

  xscale(range(-.85 1.35)) yscale(range(-.15 1.00)) ///

  graphregion(color(white)) plotregion(color(white))

* *

graph export "figures\Figure2_PCA_simple_check.png", replace width(3500)

restore

* *

* *

/********************************************************************************

Table 1. Correlations among DNAm scores

* *

Note:

  asdoc must be installed before running this block:

    ssc install asdoc, replace

********************************************************************************/

* *

asdoc pwcorr z_CellDRIFTW7 z_epiTOC2W7 z_MiAgeW7 z_PhenoAgeW7 z_GrimAgeV2W7 z_DunedinPACETT7, star(all) ///

    replace ///

    label ///

    dec(3) ///

    font(Times New Roman) ///

    save(Table1.doc) ///

    title(Table 1. Bivariate correlations among study variables)

* *

* *

* *

* *

/**********************************************************************

4. CFA MODEL CHECK

* *

The unconstrained models place the MiAge residual variance at the zero

boundary. The two reported models therefore use the same fixed residual

variance of 0.01.

**********************************************************************/

* *

sem ///

  (General -> z_CellDRIFTW7 z_epiTOC2W7 z_MiAgeW7 ///

      z_PhenoAgeW7 z_GrimAgeV2W7 z_DunedinPACETT7), ///

  variance(e.z_MiAgeW7@.01) method(ml)

* *

estimates store CFA_ONE

estat gof, stats(all)

sem, standardized

estat ic

* *

sem ///

  (Mitotic -> z_CellDRIFTW7 z_epiTOC2W7 z_MiAgeW7) ///

  (Physio -> z_PhenoAgeW7 z_GrimAgeV2W7 z_DunedinPACETT7), ///

  covariance(Mitotic*Physio) ///

  variance(e.z_MiAgeW7@.01) method(ml)

* *

estimates store CFA_TWO

estat gof, stats(all)

sem, standardized

estat ic

* *

estimates stats CFA_ONE CFA_TWO

* *

estimates restore CFA_TWO

predict double fs_mitotic fs_physio, latent

egen z_fs_mitotic = std(fs_mitotic)

egen z_fs_physio = std(fs_physio)

* *

/**********************************************************************

5. EQUAL-WEIGHT DOMAIN SCORES

**********************************************************************/

* *

egen mito_raw = rowmean(z_CellDRIFTW7 z_epiTOC2W7 z_MiAgeW7)

egen z_mitotic_domain = std(mito_raw)

* *

egen phys_raw = rowmean(z_PhenoAgeW7 z_GrimAgeV2W7 z_DunedinPACETT7)

egen z_physaging_domain = std(phys_raw)

* *

alpha z_CellDRIFTW7 z_epiTOC2W7 z_MiAgeW7, item

alpha z_PhenoAgeW7 z_GrimAgeV2W7 z_DunedinPACETT7, item

* *

pwcorr z_mitotic_domain z_physaging_domain, sig obs

pwcorr z_mitotic_domain z_fs_mitotic, sig obs

pwcorr z_physaging_domain z_fs_physio, sig obs

* *

/**********************************************************************

6. PRIMARY EXPOSURE AND INFLAMMATION MODELS

**********************************************************************/

* *

regress z_mitotic_domain z_CRP z_BMI z_AHRR_smoking z_ATS ///

  c.age_c c.age_c2 i.FemaleW7, vce(robust)

estimates store MITO_EXP

estat vif

* *

regress z_physaging_domain z_CRP z_BMI z_AHRR_smoking z_ATS ///

  c.age_c c.age_c2 i.FemaleW7, vce(robust)

estimates store PHYS_EXP

estat vif

* *

* coefplot must be installed once: ssc install coefplot, replace

coefplot ///

  (MITO_EXP, msymbol(circle) mcolor(gs7) ///

    ciopts(lcolor(gs7) lwidth(medthin))), ///

    bylabel("Panel A. Replication/mitotic") ///

  || ///

  (PHYS_EXP, msymbol(circle) mcolor(maroon) ///

    ciopts(lcolor(maroon) lwidth(medthin))), ///

    bylabel("Panel B. Early-life systemic dysregulation") ///

  ||, ///

  keep(z_CRP z_BMI z_AHRR_smoking z_ATS) ///

  order(z_CRP z_BMI z_AHRR_smoking z_ATS) ///

  coeflabels(z_CRP="CRP" z_BMI="BMI" ///

    z_AHRR_smoking="AHRR/cg05575921" ///

    z_ATS="Alcohol methylation") ///

  xline(0, lpattern(dash) lcolor(gs10)) ///

  xlabel(-0.2 "−0.2" 0 "0" 0.2 "0.2" 0.4 "0.4" 0.6 "0.6") ///

  levels(95) byopts(cols(2) note("")) legend(off) ///

  xtitle("Standardized coefficient with robust 95% CI") ///

  graphregion(color(white)) plotregion(color(white))

* *

graph export "figures\Figure3_exposure_simple_check.png", replace width(4000)

* *

/**********************************************************************

7. PRIMARY DNAmTL AND IMMUNE-CELL MODELS

**********************************************************************/

* *

regress z_mitotic_domain z_DNAmTLW7 z_CD8T_W7 z_CD4T_W7 ///

  z_NK_W7 z_Bcell_W7 z_Mono_W7 ///

  c.age_c c.age_c2 i.FemaleW7, vce(robust)

estimates store MITO_CELL

estat vif

* *

regress z_physaging_domain z_DNAmTLW7 z_CD8T_W7 z_CD4T_W7 ///

  z_NK_W7 z_Bcell_W7 z_Mono_W7 ///

  c.age_c c.age_c2 i.FemaleW7, vce(robust)

estimates store PHYS_CELL

estat vif

* *

coefplot ///

  (MITO_CELL, msymbol(circle) mcolor(gs7) ///

    ciopts(lcolor(gs7) lwidth(medthin))), ///

    bylabel("Panel A. Replication/mitotic") ///

  || ///

  (PHYS_CELL, msymbol(circle) mcolor(maroon) ///

    ciopts(lcolor(maroon) lwidth(medthin))), ///

    bylabel("Panel B. Early-life systemic dysregulation") ///

  ||, ///

  keep(z_DNAmTLW7 z_CD8T_W7 z_CD4T_W7 z_NK_W7 z_Bcell_W7 z_Mono_W7) ///

  order(z_DNAmTLW7 z_CD8T_W7 z_CD4T_W7 z_NK_W7 z_Bcell_W7 z_Mono_W7) ///

  coeflabels(z_DNAmTLW7="DNAm telomere length" ///

    z_CD8T_W7="CD8+ T cells" ///

    z_CD4T_W7="CD4+ T cells" ///

    z_NK_W7="Natural killer cells" ///

    z_Bcell_W7="B cells" ///

    z_Mono_W7="Monocytes") ///

  xline(0, lpattern(dash) lcolor(gs10)) ///

  xlabel(-0.5 "−0.5" 0 "0" 0.5 "0.5") ///

  levels(95) byopts(cols(2) note("")) legend(off) ///

  xtitle("Standardized coefficient with robust 95% CI") ///

  graphregion(color(white)) plotregion(color(white))

* *

graph export "figures\Figure4_telomere_cell_simple_check.png", replace width(4000)

* *

/**********************************************************************

8. FORMAL CROSS-DOMAIN TESTS

* *

The two regressions below omit vce(robust) because suest computes the

joint robust covariance matrix.

**********************************************************************/

* *

regress z_physaging_domain z_CRP z_BMI z_AHRR_smoking z_ATS ///

  c.age_c c.age_c2 i.FemaleW7

estimates store PHYS_EXP_SUEST

* *

regress z_mitotic_domain z_CRP z_BMI z_AHRR_smoking z_ATS ///

  c.age_c c.age_c2 i.FemaleW7

estimates store MITO_EXP_SUEST

* *

suest PHYS_EXP_SUEST MITO_EXP_SUEST, vce(robust)

* *

test [PHYS_EXP_SUEST_mean]z_CRP = [MITO_EXP_SUEST_mean]z_CRP

test [PHYS_EXP_SUEST_mean]z_BMI = [MITO_EXP_SUEST_mean]z_BMI

test [PHYS_EXP_SUEST_mean]z_AHRR_smoking = [MITO_EXP_SUEST_mean]z_AHRR_smoking

test [PHYS_EXP_SUEST_mean]z_ATS = [MITO_EXP_SUEST_mean]z_ATS

* *

regress z_physaging_domain z_DNAmTLW7 z_CD8T_W7 z_CD4T_W7 ///

  z_NK_W7 z_Bcell_W7 z_Mono_W7 ///

  c.age_c c.age_c2 i.FemaleW7

estimates store PHYS_CELL_SUEST

* *

regress z_mitotic_domain z_DNAmTLW7 z_CD8T_W7 z_CD4T_W7 ///

  z_NK_W7 z_Bcell_W7 z_Mono_W7 ///

  c.age_c c.age_c2 i.FemaleW7

estimates store MITO_CELL_SUEST

* *

suest PHYS_CELL_SUEST MITO_CELL_SUEST, vce(robust)

* *

test [PHYS_CELL_SUEST_mean]z_DNAmTLW7 = [MITO_CELL_SUEST_mean]z_DNAmTLW7

test [PHYS_CELL_SUEST_mean]z_CD8T_W7 = [MITO_CELL_SUEST_mean]z_CD8T_W7

test [PHYS_CELL_SUEST_mean]z_CD4T_W7 = [MITO_CELL_SUEST_mean]z_CD4T_W7

test [PHYS_CELL_SUEST_mean]z_NK_W7 = [MITO_CELL_SUEST_mean]z_NK_W7

test [PHYS_CELL_SUEST_mean]z_Bcell_W7 = [MITO_CELL_SUEST_mean]z_Bcell_W7

test [PHYS_CELL_SUEST_mean]z_Mono_W7 = [MITO_CELL_SUEST_mean]z_Mono_W7

* *

/**********************************************************************

9. CFA FACTOR-SCORE SENSITIVITY MODELS

**********************************************************************/

* *

regress z_fs_mitotic z_CRP z_BMI z_AHRR_smoking z_ATS ///

  c.age_c c.age_c2 i.FemaleW7, vce(robust)

estimates store FSM_EXP

* *

regress z_fs_physio z_CRP z_BMI z_AHRR_smoking z_ATS ///

  c.age_c c.age_c2 i.FemaleW7, vce(robust)

estimates store FSP_EXP

* *

regress z_fs_mitotic z_DNAmTLW7 z_CD8T_W7 z_CD4T_W7 ///

  z_NK_W7 z_Bcell_W7 z_Mono_W7 ///

  c.age_c c.age_c2 i.FemaleW7, vce(robust)

estimates store FSM_CELL

* *

regress z_fs_physio z_DNAmTLW7 z_CD8T_W7 z_CD4T_W7 ///

  z_NK_W7 z_Bcell_W7 z_Mono_W7 ///

  c.age_c c.age_c2 i.FemaleW7, vce(robust)

estimates store FSP_CELL

* *

* Cross-factor exposure tests.

regress z_fs_physio z_CRP z_BMI z_AHRR_smoking z_ATS ///

  c.age_c c.age_c2 i.FemaleW7

estimates store FSP_EXP_SUEST

* *

regress z_fs_mitotic z_CRP z_BMI z_AHRR_smoking z_ATS ///

  c.age_c c.age_c2 i.FemaleW7

estimates store FSM_EXP_SUEST

* *

suest FSP_EXP_SUEST FSM_EXP_SUEST, vce(robust)

* *

test [FSP_EXP_SUEST_mean]z_CRP = [FSM_EXP_SUEST_mean]z_CRP

test [FSP_EXP_SUEST_mean]z_BMI = [FSM_EXP_SUEST_mean]z_BMI

test [FSP_EXP_SUEST_mean]z_AHRR_smoking = [FSM_EXP_SUEST_mean]z_AHRR_smoking

test [FSP_EXP_SUEST_mean]z_ATS = [FSM_EXP_SUEST_mean]z_ATS

* *

* Cross-factor DNAmTL and immune-cell tests.

regress z_fs_physio z_DNAmTLW7 z_CD8T_W7 z_CD4T_W7 ///

  z_NK_W7 z_Bcell_W7 z_Mono_W7 ///

  c.age_c c.age_c2 i.FemaleW7

estimates store FSP_CELL_SUEST

* *

regress z_fs_mitotic z_DNAmTLW7 z_CD8T_W7 z_CD4T_W7 ///

  z_NK_W7 z_Bcell_W7 z_Mono_W7 ///

  c.age_c c.age_c2 i.FemaleW7

estimates store FSM_CELL_SUEST

* *

suest FSP_CELL_SUEST FSM_CELL_SUEST, vce(robust)

* *

test [FSP_CELL_SUEST_mean]z_DNAmTLW7 = [FSM_CELL_SUEST_mean]z_DNAmTLW7

test [FSP_CELL_SUEST_mean]z_CD8T_W7 = [FSM_CELL_SUEST_mean]z_CD8T_W7

test [FSP_CELL_SUEST_mean]z_CD4T_W7 = [FSM_CELL_SUEST_mean]z_CD4T_W7

test [FSP_CELL_SUEST_mean]z_NK_W7 = [FSM_CELL_SUEST_mean]z_NK_W7

test [FSP_CELL_SUEST_mean]z_Bcell_W7 = [FSM_CELL_SUEST_mean]z_Bcell_W7

test [FSP_CELL_SUEST_mean]z_Mono_W7 = [FSM_CELL_SUEST_mean]z_Mono_W7

* *

/**********************************************************************

10. INVERSE-PROBABILITY-OF-SELECTION WEIGHTED SENSITIVITY MODELS

**********************************************************************/

* *

regress z_mitotic_domain z_CRP z_BMI z_AHRR_smoking z_ATS ///

  c.age_c c.age_c2 i.FemaleW7 [pweight=sw_select], vce(robust)

* *

regress z_physaging_domain z_CRP z_BMI z_AHRR_smoking z_ATS ///

  c.age_c c.age_c2 i.FemaleW7 [pweight=sw_select], vce(robust)

* *

regress z_mitotic_domain z_DNAmTLW7 z_CD8T_W7 z_CD4T_W7 ///

  z_NK_W7 z_Bcell_W7 z_Mono_W7 ///

  c.age_c c.age_c2 i.FemaleW7 [pweight=sw_select], vce(robust)

* *

regress z_physaging_domain z_DNAmTLW7 z_CD8T_W7 z_CD4T_W7 ///

  z_NK_W7 z_Bcell_W7 z_Mono_W7 ///

  c.age_c c.age_c2 i.FemaleW7 [pweight=sw_select], vce(robust)

* *

/**********************************************************************

11. COMPONENT-LEVEL EXPOSURE MODELS

**********************************************************************/

* *

regress z_CellDRIFTW7 z_CRP z_BMI z_AHRR_smoking z_ATS ///

  c.age_c c.age_c2 i.FemaleW7, vce(robust)

* *

regress z_epiTOC2W7 z_CRP z_BMI z_AHRR_smoking z_ATS ///

  c.age_c c.age_c2 i.FemaleW7, vce(robust)

* *

regress z_MiAgeW7 z_CRP z_BMI z_AHRR_smoking z_ATS ///

  c.age_c c.age_c2 i.FemaleW7, vce(robust)

* *

regress z_PhenoAgeW7 z_CRP z_BMI z_AHRR_smoking z_ATS ///

  c.age_c c.age_c2 i.FemaleW7, vce(robust)

* *

regress z_GrimAgeV2W7 z_CRP z_BMI z_AHRR_smoking z_ATS ///

  c.age_c c.age_c2 i.FemaleW7, vce(robust)

* *

regress z_DunedinPACETT7 z_CRP z_BMI z_AHRR_smoking z_ATS ///

  c.age_c c.age_c2 i.FemaleW7, vce(robust)

* *

/**********************************************************************

12. LEAVE-ONE-OUT PHYSIOLOGICAL DOMAIN MODELS

**********************************************************************/

* *

egen phys_no_grim_raw = rowmean(z_PhenoAgeW7 z_DunedinPACETT7)

egen z_phys_no_grim = std(phys_no_grim_raw)

* *

regress z_phys_no_grim z_CRP z_BMI z_AHRR_smoking z_ATS ///

  c.age_c c.age_c2 i.FemaleW7, vce(robust)

* *

egen phys_no_pheno_raw = rowmean(z_GrimAgeV2W7 z_DunedinPACETT7)

egen z_phys_no_pheno = std(phys_no_pheno_raw)

* *

regress z_phys_no_pheno z_CRP z_BMI z_AHRR_smoking z_ATS ///

  c.age_c c.age_c2 i.FemaleW7, vce(robust)

* *

egen phys_no_pace_raw = rowmean(z_PhenoAgeW7 z_GrimAgeV2W7)

egen z_phys_no_pace = std(phys_no_pace_raw)

* *

regress z_phys_no_pace z_CRP z_BMI z_AHRR_smoking z_ATS ///

  c.age_c c.age_c2 i.FemaleW7, vce(robust)

* *

/**********************************************************************

13. AGE-RESIDUALIZED PCA CHECK

**********************************************************************/

* *

regress z_CellDRIFTW7 c.age_c c.age_c2

predict double r_CellDRIFT, residuals

egen zr_CellDRIFT = std(r_CellDRIFT)

* *

regress z_epiTOC2W7 c.age_c c.age_c2

predict double r_epiTOC2, residuals

egen zr_epiTOC2 = std(r_epiTOC2)

* *

regress z_MiAgeW7 c.age_c c.age_c2

predict double r_MiAge, residuals

egen zr_MiAge = std(r_MiAge)

* *

regress z_PhenoAgeW7 c.age_c c.age_c2

predict double r_PhenoAge, residuals

egen zr_PhenoAge = std(r_PhenoAge)

* *

regress z_GrimAgeV2W7 c.age_c c.age_c2

predict double r_GrimAgeV2, residuals

egen zr_GrimAgeV2 = std(r_GrimAgeV2)

* *

regress z_DunedinPACETT7 c.age_c c.age_c2

predict double r_DunedinPACE, residuals

egen zr_DunedinPACE = std(r_DunedinPACE)

* *

pca zr_CellDRIFT zr_epiTOC2 zr_MiAge ///

  zr_PhenoAge zr_GrimAgeV2 zr_DunedinPACE

* *

pwcorr zr_CellDRIFT zr_epiTOC2 zr_MiAge ///

  zr_PhenoAge zr_GrimAgeV2 zr_DunedinPACE, sig obs

* *

/**********************************************************************

14. EACH IMMUNE-CELL ESTIMATE ENTERED SEPARATELY

**********************************************************************/

* *

regress z_mitotic_domain z_CD8T_W7 z_DNAmTLW7 ///

  c.age_c c.age_c2 i.FemaleW7, vce(robust)

regress z_physaging_domain z_CD8T_W7 z_DNAmTLW7 ///

  c.age_c c.age_c2 i.FemaleW7, vce(robust)

* *

regress z_mitotic_domain z_CD4T_W7 z_DNAmTLW7 ///

  c.age_c c.age_c2 i.FemaleW7, vce(robust)

regress z_physaging_domain z_CD4T_W7 z_DNAmTLW7 ///

  c.age_c c.age_c2 i.FemaleW7, vce(robust)

* *

regress z_mitotic_domain z_NK_W7 z_DNAmTLW7 ///

  c.age_c c.age_c2 i.FemaleW7, vce(robust)

regress z_physaging_domain z_NK_W7 z_DNAmTLW7 ///

  c.age_c c.age_c2 i.FemaleW7, vce(robust)

* *

regress z_mitotic_domain z_Bcell_W7 z_DNAmTLW7 ///

  c.age_c c.age_c2 i.FemaleW7, vce(robust)

regress z_physaging_domain z_Bcell_W7 z_DNAmTLW7 ///

  c.age_c c.age_c2 i.FemaleW7, vce(robust)

* *

regress z_mitotic_domain z_Mono_W7 z_DNAmTLW7 ///

  c.age_c c.age_c2 i.FemaleW7, vce(robust)

regress z_physaging_domain z_Mono_W7 z_DNAmTLW7 ///

  c.age_c c.age_c2 i.FemaleW7, vce(robust)

* *

/**********************************************************************

15. Table A7: SINGLE MITOTIC COMPOSITE

* *

The reviewer asked whether the two-domain pattern remains when the three

highly correlated replication/mitotic scores are represented by one

composite rather than treated as three separate indicators.

**********************************************************************/

* *

* The standardized equal-weight mitotic composite was created in Section 5.

* Examine its correlations with the three physiological aging scores.

pwcorr z_mitotic_domain z_PhenoAgeW7 z_GrimAgeV2W7 z_DunedinPACETT7, sig obs

* *

* Optional Word table for the revision/supplement.

asdoc pwcorr z_mitotic_domain z_PhenoAgeW7 z_GrimAgeV2W7 z_DunedinPACETT7, ///

  star(all) replace label dec(3) font(Times New Roman) ///

  save(TableA7_MitoticComposite_Correlations.doc) ///

  title(Sensitivity analysis: mitotic composite and physiological aging scores)

* *

/**********************************************************************

16. Figure A1: FOUR-VARIABLE PCA

* *

Repeat PCA using one mitotic composite plus PhenoAge, GrimAgeV2, and

DunedinPACE. This directly tests whether the separation remains after

collapsing CellDRIFT, EpiTOC2, and MiAge into a single measure.

**********************************************************************/

* *

pca z_mitotic_domain z_PhenoAgeW7 z_GrimAgeV2W7 z_DunedinPACETT7

* *

* Convert the first two PCA eigenvectors to correlations/loadings.

matrix V4 = e(L)

matrix EV4 = e(Ev)

matrix LOAD4 = V4[1...,1..2]

* *

forvalues i = 1/4 {

  matrix LOAD4[`i',1] = LOAD4[`i',1]*sqrt(EV4[1])

  matrix LOAD4[`i',2] = LOAD4[`i',2]*sqrt(EV4[1,2])

}

* *

matrix colnames LOAD4 = load1 load2

matrix rownames LOAD4 = MitoticComposite PhenoAge GrimAgeV2 DunedinPACE

matrix list LOAD4

matrix list EV4

* *

* Plot the four-variable PCA for the reviewer/supplement.

preserve

clear

svmat double LOAD4, names(col)

* *

gen str20 score = ""

replace score = "Mitotic composite" in 1

replace score = "PhenoAge" in 2

replace score = "GrimAgeV2" in 3

replace score = "DunedinPACE" in 4

* *

gen byte domain = 1 in 1

replace domain = 2 in 2/4

* *

gen double x0 = 0

gen double y0 = 0

* *

twoway ///

  (pcarrow y0 x0 load2 load1, ///

    lcolor(gs6) mcolor(gs6) lwidth(thin)) ///

  (scatter load2 load1 if domain == 1, ///

    msymbol(triangle) msize(medlarge) mcolor(red) ///

    mlabel(score) mlabpos(3) mlabsize(medsmall) ///

    mlabcolor(red) mlabgap(2)) ///

  (scatter load2 load1 if domain == 2, ///

    msymbol(circle) msize(medlarge) mcolor(navy) ///

    mlabel(score) mlabpos(3) mlabsize(medsmall) ///

    mlabcolor(navy) mlabgap(2)), ///

  xline(0, lpattern(dash) lcolor(gs10)) ///

  yline(0, lpattern(dash) lcolor(gs10)) ///

  xscale(range(-.22 1.05)) ///

  xlabel(-0.2 "−0.2" 0 "0" 0.2 "0.2" 0.4 "0.4" ///

    0.6 "0.6" 0.8 "0.8" 1.0 "1.0") ///

  ylabel(0 "0" 0.2 "0.2" 0.4 "0.4" 0.6 "0.6" ///

    0.8 "0.8" 1.0 "1.0") ///

  legend(order(1 "Loading" ///

        2 "Replication/mitotic composite" ///

        3 "Early-life systemic dysregulation") ///

    position(2) ring(0) cols(1) ///

    size(small) region(lcolor(none))) ///

  xtitle("Principal Component 1") ///

  ytitle("Principal Component 2") ///

  graphregion(color(white)) ///

  plotregion(color(white))

* *

graph export "figures\Figure A1_MitoticComposite_PCA.png", ///

  replace width(3500)

* *

/**********************************************************************

17. CLEAN UP

**********************************************************************/

* *

capture erase "_included_ids_for_simple_check.dta"

capture erase "_selection_weights_for_simple_check.dta"

* *

log close
